# The two-component system CpxAR is required for the high potassium stress survival of *Actinobacillus pleuropneumoniae*

**DOI:** 10.3389/fmicb.2023.1259935

**Published:** 2023-09-26

**Authors:** Jiajia Wan, Rui Zhang, Yizhen Jia, Tingting Xie, Lu Dai, Qing Yao, Wendie Zhang, Huasong Xiao, Xuejun Gao, Jing Huang, Weicheng Bei, Feng Liu

**Affiliations:** ^1^College of Animal Sciences, Yangtze University, Jingzhou, Hubei, China; ^2^College of Arts and Sciences, Yangtze University, Jingzhou, Hubei, China; ^3^State Key Laboratory of Agricultural Microbiology, College of Veterinary Medicine, Huazhong Agricultural University, Wuhan, Hubei, China

**Keywords:** *Actinobacillus pleuropneumoniae*, two-component system, CpxAR, high-K^+^ stress, cell division, *ftsEX*

## Abstract

**Introduction:**

*Actinobacillus pleuropneumoniae* is an important respiratory pathogen, which can cause porcine contagious pleuropneumonia and lead to great economic losses to worldwide swine industry. High potassium is an adverse environment for bacteria, which is not conducive to providing turgor pressure for cell growth and division. Two-component system CpxAR is an important regulatory system of bacteria in response to environmental changes, which is involved in a variety of biological activities, such as antibiotic resistance, periplasmic protein folding, peptidoglycan metabolism and so on.

**Methods:**

However, little is known about the role of CpxAR in high potassium stress in *A. pleuropneumoniae*. Here, we showed that CpxAR is critical for cell division of *A. pleuropneumoniae* under high potassium (K^+^) stress.

**Results:**

qRT-PCR analysis found that CpxAR positively regulated the cell division genes *ftsEX.* In addition, we also demonstrated that CpxR-P could directly bind the promoter region of the cell division gene *ftsE* by EMSA.

**Discussion:**

In conclusion, our results described a mechanism where CpxAR adjusts *A. pleuropneumoniae* survival under high-K^+^ stress by upregulating the expression of the cell division proteins FtsE and FtsX. These findings are the first to directly demonstrate CpxAR-mediated high-K^+^ tolerance, and to investigate the detailed molecular mechanism.

## Introduction

*Actinobacillus pleuropneumoniae* is the aetiological agent of porcine pleuropneumonia, a serious respiratory disease with high morbidity, high lethality and substantial economic losses in the worldwide swine industry (Pattison et al., 1957; Frey, 1995; Sassu et al., 2018). Currently, 19 serovars of *A. pleuropneumoniae* have been identified based on the antigenic diversity of the capsular polysaccharides and lipopolysaccharides (Stringer et al., 2021; Scherrer et al., 2022). To successfully invade the host, *A. pleuropneumoniae* regulates the expression of its metabolism and virulence-related genes to adapt to adverse environments, such as cold, heat and osmotic.

In bacteria, two-component system (TCS) is a fine signal network to sense any change, and regulates the expression of bacterial effectors for adaptation and survival (Zschiedrich et al., 2016). To increase their adaptability, *A. pleuropneumoniae* has encoded five two-component systems (TCS) to detect and respond to diverse environments, such as CpxAR, QseBC, PhoBR, ArcAB and NarPQ (Xu et al., 2008). The CpxAR TCS is found in many bacteria, consisting of CpxA (histidine kinase) and CpxR (transcriptional regulatory protein), and has been implicated in envelope stress responses. In *A. pleuropneumoniae*, the CpxAR plays an important role in heat stress, cold stress, biofilm formation, capsule synthesis, and pathogenesis (Li et al., 2018; Yan et al., 2020; Yao et al., 2022; Liu et al., 2022a,b).

In *A. pleuropneumoniae*, the CpxAR modulates biofilm formation and virulence by regulating the expression of the *pgaABCD* operon through *rpoE* (Li et al., 2018). CpxAR is also found to mediate *A. pleuropneumoniae* stress resistance and virulence by activating the expression of WecA, which is essential for O-antigen biosynthesis (Yan et al., 2020). Moreover, CpxAR responds to heat stress by suppressing the expression of the type IV pilin ApfA, which is prone to misfolding and aggregation and therefore reduces bacterial survival under heat stress (Liu et al., 2022a). In addition, we demonstrated previously that CpxAR contributes to virulence by upregulating the expression of the polysaccharide capsule export system CpxDCBA (Liu et al., 2022b).

Potassium (K^+^) is the major cation in bacterial cytoplasm, and essential for maintaining ion homeostasis which is important for regulating pH and membrane potential (Ballal et al., 2007). But, excessive K^+^ is cytotoxic to bacteria, affecting turgor pressure which can lead to disturbances in cell growth and division (Sweet et al., 2021). However, most bacteria have evolved several K^+^-translocating systems to maintain K^+^ homeostasis, such as TrkAH, KtrAB, KtrCD, KdpFABC, KimA, Kup, Kef, YjbQ and KhtSTU (Stautz et al., 2021).

Schmidt and colleagues found that FtsE and FtsX are essential for cell division (Schmidt et al., 2004). In addition, previous studies have shown that FtsE contributes to the translocation of K-pump to the cell membrane including KdpA, TrkH, and Kup (Ukai et al., 1998; Mir et al., 2015). In *E. coli*, disruption of FtsE function exhibited growth defect under high-K^+^ stress (Ukai et al., 1998).

In this study, we showed that CpxAR contributes to *A. pleuropneumoniae* growth and cell division under high-K^+^ stress. In addition, we confirmed that CpxAR directly activates the expression of cell division genes *ftsEX*. Taken together, our study describes a new mechanism by which CpxAR enables *A. pleuropneumoniae* to adjust its survival by up-regulating the expression of *ftsEX* under high-K^+^ stress.

## Materials and methods

### Strains, plasmid, and growth conditions

In this study, *A. pleuropneumoniae* strain S4074 was used as a representative strain, which was grown at 37°C in tryptic soy broth (TSB; BD, United States) added with 10% FBS and 10% (vol/vol) nicotinamide adenine dinucleotide (NAD; Biofroxx, Germany). The wild-type S4074 and in-frame mutant strain ∆*cpxRA* were donated by Prof. Weicheng Bei (Li et al., 2018). *Escherichia coli β2155* was cultured at 37°C in Luria-Bertani (LB) with 50 μg/mL diaminopimelic acid (DAP; Sigma, USA). The bacterial strains used in this study are listed in Table 1, and primers are described in Table 2. The recombinant plasmid pJFF224-P*ftsEX* with IPTG-inducible promoter was electrically transferred to the ∆*cpxRA* mutant strains to regulate the *ftsEX* gene expression under the different concentrations of IPTG.

**Table 1:** Bacterial strains and plasmids used in this study.

**Strains/plasmids**	**Characteristics**	**Source/reference**
*A. pleuropneumoniae*		
S4074	*A. pleuropneumoniae* reference strain of serovar 1; WT strain	From Prof. Weicheng Bei
Δ*cpxRA*	*A. pleuropneumoniae* 4,074 *cpxRA*-deletion mutant	From Prof. Weicheng Bei
CΔ*cpxRA*	Complemented strain of Δ*cpxRA*; Cm^r^	From Prof. Weicheng Bei
*E. coli*		
DH5α	Cloning host for recombinant vector	Takara
BL21	Expression protein for recombinant vector	Takara
*Plasmid*		
pET-30a	Expression vector; Kan^r^	Novagen
pET30a-*cpxR*	pET-30a carrying *cpxR* gene	This study
pJFF224-XN	*E. coli*-APP shuttle vector: RSF1010 replicon; mob oriV, Cm^r^	From Prof. Weicheng Bei
p*ftsE*-*gfp*	pJFF224-XN carrying the *ftsE*-*gfp* fusions	This study

Cm^r^, Chloramphenicol resistance; Amp^r^, Ampicillin resistance; Kan^r^, Kanamycin resistance.

**Table 2:** Primers used in this study.

Primer	Sequence (5′–3′) a	Product	Use
*ftsE-F/R*	CCGTAGTACCCGCCTGATTA	219	Detection the transcription of *ftsE*
	AAGCGCGTATTGCCTTAGAG		
*ftsX-F/R*	CGGTATAAAGGAACGGACGA	208	Detection the transcription of *ftsX*
	GATAACGGCTGGTTGGAAAA		
*ftsY-*F*/R*	CGTCGTCGGCATACCTAAAT	235	Detection the transcription of *ftsY*
	CGAGCTTGAAACGGAAAAAG		
*ftsL-*F*/R*	ATGGCAAGTAATGAACGTTATCCGC	287	Detection the transcription of *ftsL*
	TTTTTTATCGACTGTAAACCGAA		
*ftsI-*F*/R*	GTGGAAGGTTATCGTGTGGC	195	Detection the transcription of *ftsI*
	CGAGAATAACGGCGCAGAA		
*ftsW-*F*/R*	TCGTATTGATTTTCGGGCGC	153	Detection the transcription of *ftsW*
	GTTTGGTGCGCATTTCATCG		
*ftsQ-*F*/R*	TGCTGTACGGACCTGATACC	169	Detection the transcription of *ftQ*
	TCGCCACGACCTAATCTGAG		
*FtsZ-*1*-*F*/R*	TTGCCGATGTGAAAACCGTT	154	Detection the transcription of *ftsZ*
	AAGATACCTTTCGCACCGGA		
*FtsZ-*2*-*F*/R*	TTTTGTTGCTGTTCTGCCGA	158	Detection the transcription of *ftsZ*
	ATCGGTATTGGCGAATCAGG		
*ftsB-*F*/R*	GTTGGAGCGATTATCAAGAAGC	161	Detection the transcription of *ftsB*
	CACCATCTCACGTTCGAAGC		
*ftsA-*F*/R*	TTTTGTGCTTACGGGTGGTG	200	Detection the transcription of *ftsA*
	TTTCGCCATTGCCCTTTTCA		
*ZipA-*F*/R*	GCGGTTGCGTTTGATATTGC	217	Detection the transcription of *ZipA*
	CAGACAACAGGACAACACGG		
*ftsH-*F*/R*	AGTCCTTTGGGTAGTGGTCG	208	Detection the transcription of *ftsH*
	CATCGGCATTACGGTTTGGT		
*ftsK-*F*/R*	TTACCGGCGTGATTAAAGCG	222	Detection the transcription of *ftsK*
	CTCTTGCACGCCAGTTATCC		
16SrRNA-F*/R*	CCATGCCGCGTGAATGA	58	Detection the transcription of 16SrRNA
	TTCCTCGCTACCGAAAGAACTT		
*ftsE*-EMSA-F*/R*	CGCCTTTATAAGCTTTACTTACATT	151	Amplification of *ftsE* promoter region for EMSA
	TTATTCGAACACGAAGAATAGCAAT		
*ftsY*-EMSA-F*/R*	GTATGTTGTTCGGTTTGGA	303	Amplification of *ftsY* promoter region for EMSA
	TTTACTCGGTCGGTGGT		
*rpoE*-EMSA-F*/R*	TAAAAAGATAAGATAAGCGGTC	273	Amplification of *rpoE* promoter region for EMSA
	AGTGTGTAACAAAAATGAAAAGT		
*rpoD*-EMSA-F*/R*	GCGGAAGAAAAGCAAGAGTTGGTCA	151	Amplification of *rpoD* promoter region for EMSA
	TCCATAATTGTATCCGTTTTGTGTG		
*ftsEX-F/R*	CCGCTCGAGACACGAAGAATAGCAATGATTACAA	1737	Amplification of *ftsEX* genes for GFP-*ftsEX* plasmid
	AGCAAGGTCATCTCAGAAATGGTACCGTATTTGGAACCGATTCGGAAT		
*GFP-F/R*	AGCAAGGTCATCTCAGAAATGGTACCAGTAAAGGAGAAGAACTTTTCAC	2,257	Amplification of GFP gene for GFP-*ftsEX* plasmid
	AAGGAAAAAAGCGGCCGCTTATTTGTATAGTTCATCCATGCC		
IPTG-*ftsEX*-F*/R*	CGGGGTACC AATAGTAGAAAAGGTAAACATAATG	1,650	Expression of *ftsEX* with the inducing of IPTG
	CGGGGTACC AATAGTAGAAAAGGTAAACATAATG		

### SEM

For scanning electron microscopy (SEM), wild-type S4074 and its mutant derivatives were harvested after they were cultured in TSB medium supplemented with 10% FBS and 10% NAD with or without the supplementation of 0.3 M KCl. Bacterial cells were fixed with 2.5% glutaraldehyde and deposited onto copper grids (200 mesh; Zhongjingkeyi, China). The copper grids were air-dried, mounted on the sample stub and coated with gold. Subsequently, strains were observed by the SEM (VEGA3; TESCAN, Czech) and the bacterial length was measured by Image J (NIH, United States).

### RNA extraction, quantitative RT-PCR, and RT-PCR

WT and ∆*cpxRA* mutant strains were cultivated at 37°C shaking in TSB supplemented with 10% FBS and 10% NAD to an OD600 of 0.6, centrifuged (3,000 g, 5 min) to gather cells. Total RNA of strains was extracted using Total RNA Extractor kit (Sangon Biotech, China) according to the manufacturer’s instruction. DNA impurity was eliminated using DNase I kit (Vazyme, China) according to instruction. To determine the quality, the RNA was electrophoresed on a 1% agarose gel. cDNA was synthesized using reverse transcriptase from Vazyme. Quantitative Real-Time PCR was performed using Real-Time Quantitative PCR detecting System and SYBR qPCR Mix (Vazyme, China). 16S RNA was used as the endogenous control to normalize expression of target genes. The 2^−ΔΔCt^ method was used to calculate and analyze relative expression level of mRNA (Livak and Schmittgen, 2001). RT-PCR across the *cdd*-*ftsX*, *ftsX*-*ftsE*, *ftsE*-*ftsY*, and *ftsY*-*rsmD* junctions was performed as previously described (Liu et al., 2022a).

### Construction of promoter-gfp repoter strains and promoter analyses

A *ftsE*-*gfp* fusion containing the *ftsE* promoter region and *gfp* gene, was cloned into the Xho I and Not I sites on the pJFF224-XN plasmid. Then, the *ftsE*-*gfp* reporter plasmid was electroporated into the wild-type S4074 and the ∆*cpxAR* mutant strain. *A. pleuropneumoniae* strains harboring a *ftsE*-*gfp* reporter plasmid were grown to an OD600 of 0.6 in TSB medium with or without the supplementation of 0.3 M KCl. The cells were harvested and resuspended in 1 mL of 10 mM PBS. Luminescence was measured in the Spectramax iD3 microplate reader with excitation at 485 nm and emission at 535 nm.

### Expression and purification of His-CpxR

The purification of CpxR was conducted as previously described (Liu et al., 2022b). The *E.coli* BL21 containing the expression vector pET30a-CpxR was used to express His-CpxR protein. Expression of His-CpxR was induced by IPTG (0.5 mM) at 16°C overnight, and bacteria were harvested by centrifuging at 12000 rpm for 10 min. The cell pellet was resuspended in binding buffer (50 mM Na_3_PO_4_, pH 7.4, 500 mM NaCl, 30 mM imidazole), and lysed by sonication. The soluble fraction of the lysate was added to nickel-nitrilotriacetic acid (Ni-NTA) agarose column and mixed on Multipurpose Shaker QB-206 for 3 h. After being washed with binding buffer, the His-CpxR protein was eluted with elution buffer (20 mM Tris–HCl, pH 7.4, 500 mM NaCl, 500 mM imidazole). The purified His-CpxR was then stored at −80°C until use.

### Electrophoretic mobility shift assay

The promoter regions of target genes were amplified from the *A. pleuropneumoniae* S4074 genome and tagged with biotin using a EMSA Probe Biotin Labeling Kit (Beyotime, China). CpxR protein was phosphorylated by acetyl phosphate (Sigma, United States) (Pogliano et al., 1997). Biotin-labeled DNA probes (1 μM) were incubated at room temperature for 20 min with 0–4 pmol CpxR protein in binding buffer (Beyotime, China). The reaction mixtures were analyzed by 4% non-denaturing polyacrylamide gel electrophoresis and transferred to nylon membrane. Next, the band was detected using a Chemiluminescent EMSA Kit (Beyotime, China).

### Statistical analysis

Experimental data were analyzed by Student’s two-tailed t-tests using GraphPad Prism 7.0 (GraphPad lnc.), and shown as mean ± standard deviation (SD). Statistical significance was assumed at a *p* value of <0.05.

## Results

### Deletion of the cpxRA genes decreases the growth of *Actinobacillus pleuropneumoniae* under high-K^+^ stress

**Figure 1:**
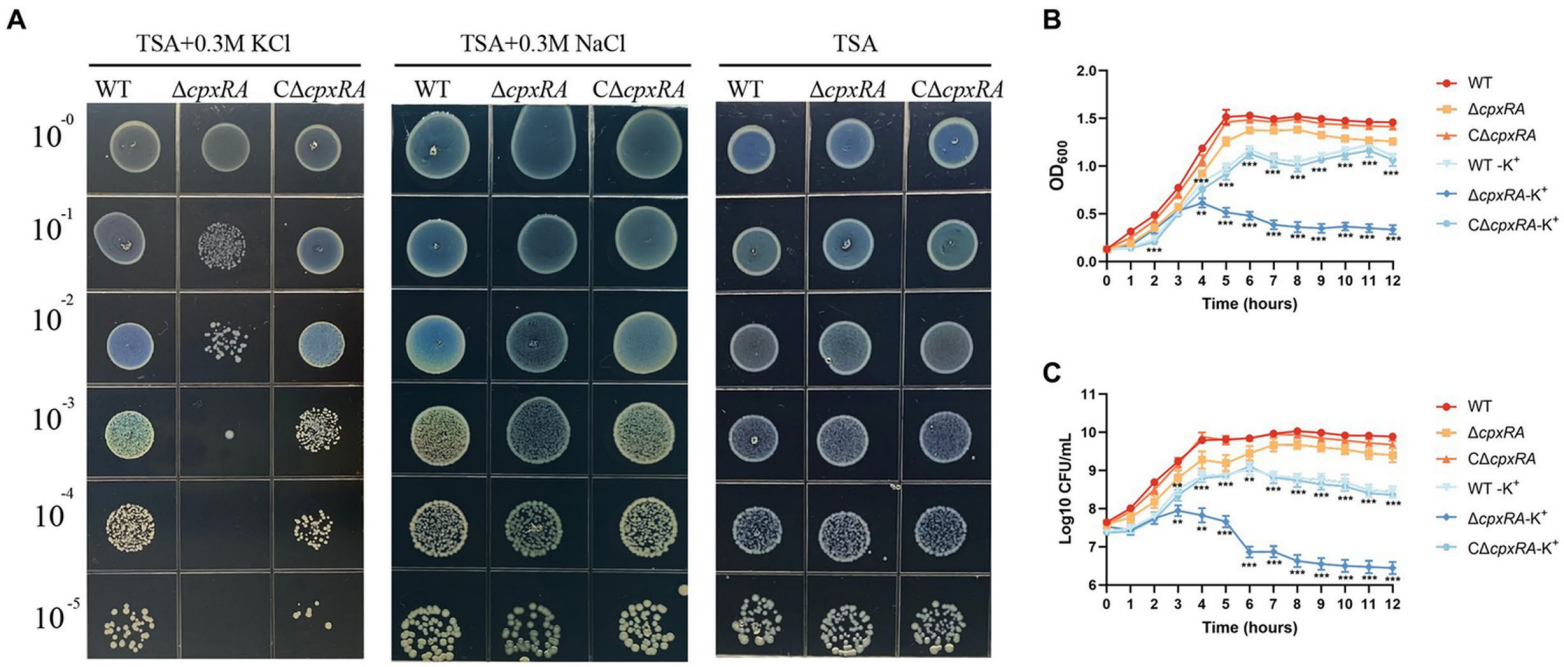
CpxAR is required for growth under high-K^+^ stress. The growth traits of the WT, ∆*cpxRA* and C∆*cpxRA* strains with or without 0.3 M K^+^ or 0.3 M Na^+^ were monitored by measurement of spotting on TSA plates **(A)**, OD600 **(B)**, and viable cell counts **(C)**. **p < 0.01. ***p < 0.001.

To explore the role of CpxAR in bacterial adaptation to osmotic stress in *A. pleuropneumoniae*, we tested the growth traits of the wild-type S4074 and its *cpxRA* mutant strain grown in solid or liquid medium with or without the supplementation of 0.3 M KCl or 0.3 M NaCl. When the cells were grown in solid medium, the growth of the ∆*cpxRA* strain was markedly reduced with the supplementation of 0.3 M KCl, but similar to that of the WT strain with the supplementation of 0.3 M NaCl or normal medium (Figure 1A). Optical density and colony forming units showed that the growth rate of the ∆*cpxRA* strain was significantly lower than that of the WT strain when grown with the supplementation of 0.3 M KCl (Figures 1B,C). These findings suggested that the growth defect of the mutant strain ∆*cpxRA* was significantly increased compared with that of WT and C∆*cpxRA* strains under high-K^+^ stress. These results suggested that CpxAR contributes to *A. pleuropneumoniae* survival under high potassium stress.

### Deletion of cpxRA affects the cell division of *Actinobacillus pleuropneumoniae* under high-K^+^ stress

**Figure 2:**
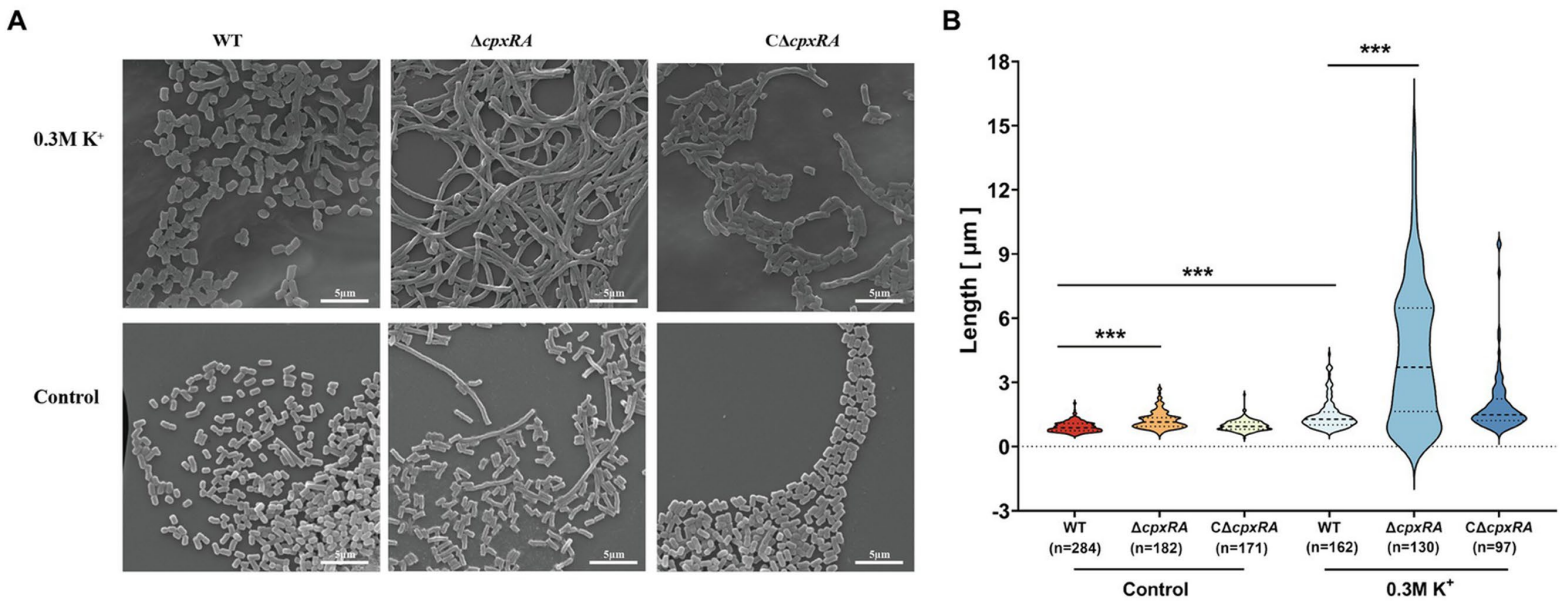
CpxAR impacts cell division under high-K^+^ stress. **(A)** Observation of bacterial length in the WT, ∆*cpxRA*, and C∆*cpxRA* strains grown with or without 0.3 M K^+^ by SEM. **(B)** Bacterial length measured for the WT, ∆*cpxRA* and C∆*cpxRA* strains grown with or without 0.3 M K^+^. The number of bacteria measured is shown in brackets. ***p < 0.001.

To further investigate how the inactivation of *cpxRA* genes affected the growth of *A. pleuropneumoniae* under high-K^+^ stress, we used scanning electron microscope (SEM) to observe the bacterial morphology of the WT, ∆*cpxRA* and C∆*cpxRA* strains when they were grown with or without the supplementation of 0.3 M KCl. When grown with the supplementation of 0.3 M KCl, the cell length of ∆*cpxRA* strain showed a 2-fold increase on average compared to the wild-type and C∆*cpxRA* strains (Figures 2A,B). In addition, we found that the cell length of ∆*cpxRA* strain was also longer than that of the wild-type and C∆*cpxRA* strains without K^+^ stress, but the difference was much smaller than that under K^+^ stress (Figures 2A,B). Together, these findings suggested that CpxAR regulates cell division to help *A. pleuropneumoniae* cope with potassium stress.

### CpxAR regulates the expression of cell division genes ftsE and ftsX under high-K^+^ stress

**Figure 3:**
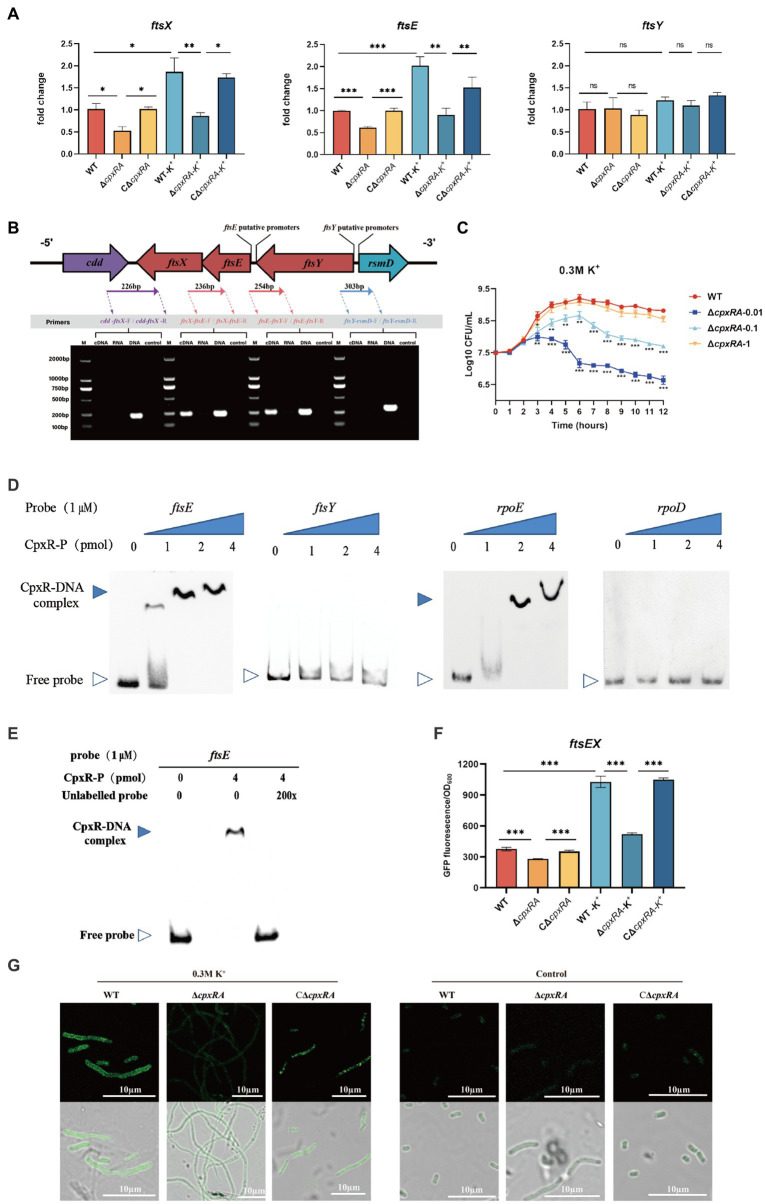
CpxAR regulates the expression of cell division genes *ftsE* and *ftsX*. **(A)** qRT-PCR analysis of *ftsE*, *ftsX*, and *ftsY* in WT, ∆*cpxRA*, and C∆*cpxRA* strains. **(B)** Schematics of organization of genes encoding the cell division proteins. Transcriptional characteristics of the *ftsE*, *ftsX*, and *ftsY* genes measured by RT-PCR. Lane 1, cDNA; lane 2, RNA; lane 3, DNA; and lane 4, negative control. **(C)** The growth analysis of strains expressing *ftsEX* with IPTG at indicated concentrations. **(D)** EMSA analysis of the binding of phosphorylated CpxR to the promoters of *ftsE*, *ftsY*, *rpoE*, and *rpoD*. *rpoE* and *rpoD* served, respectively, as positive and negative control. **(E)** EMSA analysis of the binding of purified CpxR to *ftsE* with or without unlabeled DNA probe. The promoter activity of *ftsE* gene in the WT and ∆*cpxRA* strains was analized by fluorescent microscopy **(F)** and measurement of relative fluorescence units **(G)**. *p < 0.05. **p < 0.01. ***p < 0.001.

To gain insight into the mechanism of CpxAR affecting the cell division of *A. pleuropneumoniae*, we compared the transcript levels of cell division genes in the WT and ∆*cpxRA* strains by qRT-PCR. As shown in Figure 3A, the relative transcript levels of *ftsE* and *ftsX* were significantly downregulated in the ∆*cpxRA* strain with or without the supplementation of 0.3 M KCl, but *ftsY* was not (Figure 3A). However, there were no significant changes in the cell division genes *ftsL*, *ftsI*, *ftsW*, *ftsQ*, *ftsZ*, *ftsB*, *ftsA*, *zipA*, *ftsH*, and *ftsK* in the ∆*cpxRA* strain compared with the WT strain (Supplementary Figure S1).These results suggested that CpxAR regulates the expression of the cell division genes *ftsE* and *ftsX* in *A. pleuropneumoniae*.

The cell division genes *ftsY*, *ftsE*, and *ftsX* are adjacent on the *A. pleuropneumoniae* chromosome (Figure 3B). To characterize the *ftsYEX* locus, we performed RT-PCR across the *cdd*-*ftsX*, *ftsX*-*ftsE*, *ftsE*-*ftsY*, and *ftsY*-*rsmD* junctions. The RT-PCR analysis indicated that the *ftsY*, *ftsE*, and *ftsX* genes are co-transcribed as a single mRNA and comprise an operon. To verify whether the *ftsEX* operon contributes to *A. pleuropneumoniae* survival under high-K^+^ stress, the growth of ∆*cpxRA*/P*ftsEX* was assayed. When the expression of FtsE and FtsX increased with the increase of IPTG concentration, the growth defects of the ∆*cpxRA* strain were significantly rescued (Figure 3C). These observations indicated that the *ftsEX* operon plays an important role in *A. pleuropneumoniae* response to high-K^+^ stress.

Here, the prediction results (BDGP)^1^ showed that the *ftsYEX* operon has two putative promoter regions, respectively, located upstream of *ftsY* and *ftsE*. To explore the mechanism by which CpxR regulates the *ftsYEX* operon expression, we tested whether CpxR-P binds to the promoter regions of *ftsY* and *ftsE* using gel shift analysis. EMSA analysis showed that CpxR was capable to bind with the promoter regions of *ftsE* and *rpoE* (positive control), but not to the promoter regions of *ftsY* and *rpoD* (negative control) (Figure 3D). In addition, EMSAs showed that phosphorylated CpxR did not bind to the promoter sequence of *ftsE* when 200X of unlabeled probes were added (Figure 3E). To examine the link between CpxR and the expression of the *ftsEX* operon, the GFP reporter strains for *ftsE* gene promoter were constructed. As shown in Figures 3F,G, fluorescence intensities and confocal microscopy analysis showed that the transcrition activity of the promoter was significantly decreased in ∆*cpxRA* strain with or without high-K^+^ stress. Together, these data demonstrated that CpxAR directly regulates the expression of *ftsEX* operon.

## Discussion

The two-component system is an important group of signal transduction systems in bacteria that interacts with sudden stimulus and responds accordingly to survive in adverse environment (Zhao et al., 2022). Prototypical TCS is composed of a membrane-bound sensor kinase and a cytoplasmic response regulator (Eguchi and Utsumi, 2008). In these systems, the histidine kinase will autophosphorylate when sensing a stimulus, and transfers the phosphoryl group to the response regulator, which then regulates the expression of specific genes by binding these promoters. Previous studies showed that CpxAR plays multiple regulatory roles in *A. pleuropneumoniae* virulence, biofilm formation and stress resistance (Yan et al., 2020). However, the function of the CpxAR system adaptation to other surrounding stresses remains unknown.

Potassium is the major cation in the cytoplasm for bacteria, which is essential for providing turgor pressure for cell growth and division (Sweet et al., 2021). When external K^+^ concentrations are high, bacteria adjust their survival by maintaining cellular K^+^ homeostasis. However, the mechanism of bacterial survival under high-K^+^ stress requires further exploration. In this study, the growth rate of WT strain under K+ stress was significantly lower than that of strain without K+ stress, indicating that high-K^+^ stress inhibits cell growth and division of *A. pleuropneumoniae*. Furthermore, we showed that the CpxAR system plays an important role in growth and division of *A. pleuropneumoniae* during high-K^+^ stress.

FtsEX is a putative ABC transporter type complex that facilitates the assembly of division proteins and other proteins to the cytoplasmic membrane and is thought to play an important role in cell division (Schmidt et al., 2004; Reddy, 2007). In *E. coli*, disruption of the *ftsE* gene prevents the localization of the K^+^-pump proteins to the inner membrane (Ukai et al., 1998). In the present study, qRT-PCR analysis showed that cell division genes *ftsE* and *ftsX* are controlled by CpxAR in *A. pleuropneumoniae*. Indeed, it seemed that FtsE and FtsX are related to the mechanism of CpxAR-mediated potassium stress. In *E. coli*, cell division genes *ftsY*, *ftsE*, and *ftsX* comprise an operon (Gill et al., 1986), which is consistent with our RT-PCR analysis in *A. pleuropneumoniae*. But, we found that the *ftsYEX* operon in *A. pleuropneumoniae* contains two promoter regions by sequence analysis, oneupstream of *ftsY* and the other upstream of *ftsE*.

Previous studies have shown that CpxR can regulate the transcription of a wide range of genes by directly binding to their promoter regions (De Wulf et al., 2002; Vogt and Raivio, 2012). Here, EMSA analysis showed that CpxR can directly interact with *ftsE* promoter. Combined with qRT-PCR analysis, these results confirmed that the *ftsEX* operon is directly and positively regulated by the CpxAR system.

**Figure 4:**
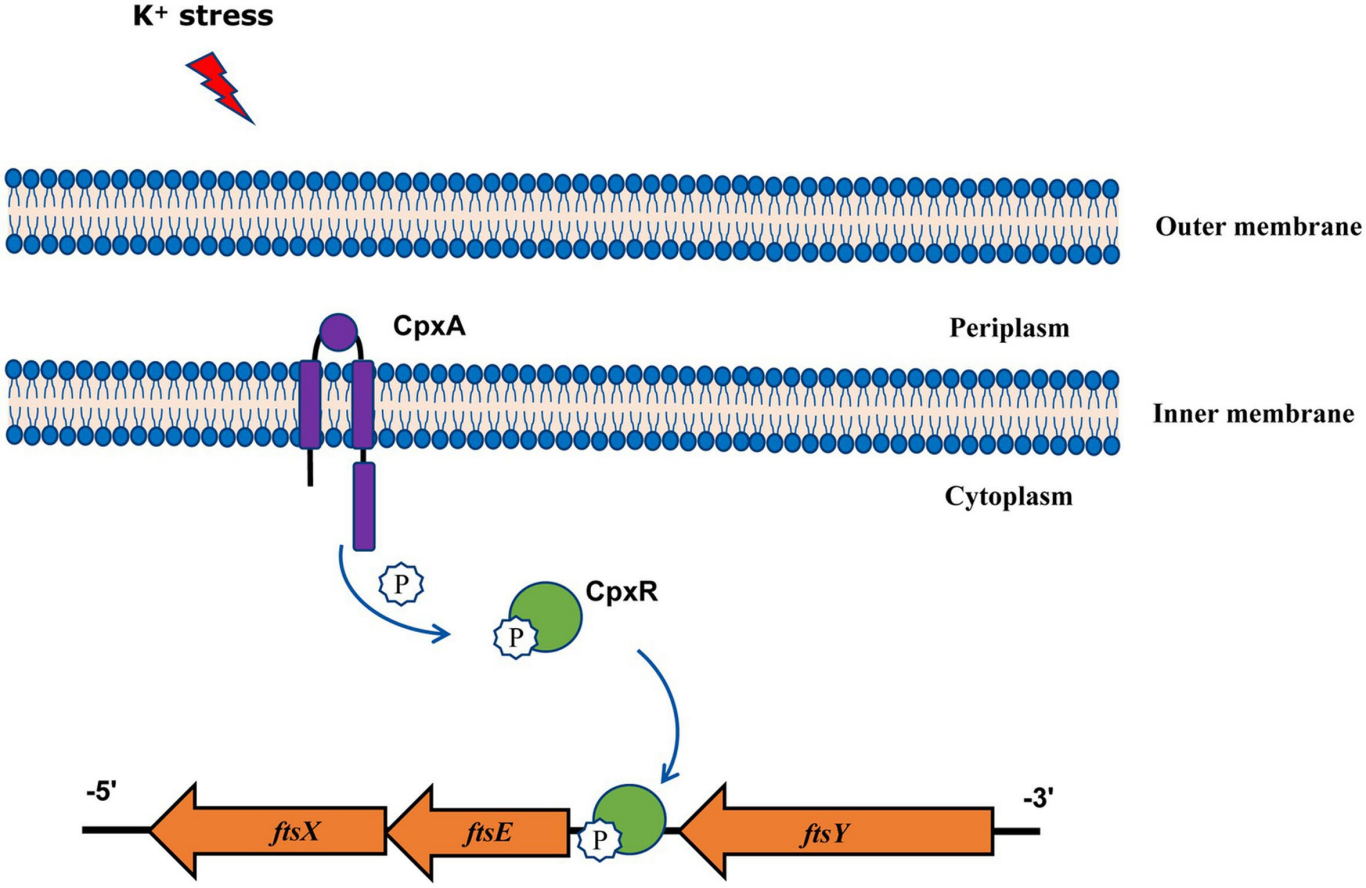
Model of CpxAR-*ftsEX* regulatory circuit in *A. pleuropneumoniae*. The high-K^+^ stress activates the TCS CpxAR which directly induces expression of the *ftsEX* operon.

In summary, our findings gain new and important insights into the function of *A. pleuropneumoniae* CpxAR in response to extreme environments, and show that the CpxAR system directly regulates cell division genes to maintain potassium homeostasis (Figure 4). Because the CpxAR system is ubiquitous in many bacteria, our study contribute to the understanding of bacterial environmental adaptability. Future studies will aim to identify more CpxR-regulated genes, which could expand the knowledge of CpxAR function.

## Data availability statement

The original contributions presented in the study are included in the article/Supplementary material, further inquiries can be directed to the corresponding author.

## Author contributions

JW: Data curation, Investigation, Project administration, Software, Writing – original draft. RZ: Investigation, Writing – original draft. YJ: Investigation, Writing – original draft. TX: Investigation, Writing – original draft. LD: Investigation, Writing – original draft. QY: Investigation, Writing – original draft. WZ: Investigation, Writing – original draft. HX: Investigation, Writing – original draft. XG: Software, Supervision, Writing – original draft. JH: Writing – review & editing. WB: Funding acquisition, Resources, Supervision, Writing – original draft. FL: Conceptualization, Formal analysis, Funding acquisition, Methodology, Supervision, Writing – original draft, Writing – review & editing.

## Funding

The author(s) declare financial support was received for the research, authorship, and/or publication of this article. This research was supported by the National Natural Science Foundation of China (32002252) and National Key Research and Development Program of China (2022YFD1800905).

## Conflict of interest

The authors declare that the research was conducted in the absence of any commercial or financial relationships that could be construed as a potential conflict of interest.

## Correction note

A correction has been made to this article. Details can be found at: 10.3389/fmicb.2025.1645185.

## Publisher’s note

All claims expressed in this article are solely those of the authors and do not necessarily represent those of their affiliated organizations, or those of the publisher, the editors and the reviewers. Any product that may be evaluated in this article, or claim that may be made by its manufacturer, is not guaranteed or endorsed by the publisher.

## Footnotes

^1^ https://www.fruitfly.org/seq_tools/promoter.html
