# Correction: The two-component system CpxAR is required for the high potassium stress survival of *Actinobacillus pleuropneumoniae*

**DOI:** 10.3389/fmicb.2025.1645185

**Published:** 2025-07-09

**Authors:** Jiajia Wan, Rui Zhang, Yizhen Jia, Tingting Xie, Lu Dai, Qing Yao, Wendie Zhang, Huasong Xiao, Xuejun Gao, Jing Huang, Weicheng Bei, Feng Liu

**Affiliations:** ^1^College of Animal Sciences, Yangtze University, Jingzhou, Hubei, China; ^2^College of Arts and Sciences, Yangtze University, Jingzhou, Hubei, China; ^3^State Key Laboratory of Agricultural Microbiology, College of Veterinary Medicine, Huazhong Agricultural University, Wuhan, Hubei, China

**Keywords:** *Actinobacillus pleuropneumoniae*, two-component system, CpxAR, high-K^+^ stress, cell division, *ftsEX*

In the published article, there was an error in the legend for **Figure 1** as published. The concentrations of potassium ions and sodium ions added in TSA were reported incorrectly. The corrected legend appears below.

Figure 1 CpxAR is required for growth under high-K^+^ stress. The growth traits of the WT, Δ*cpxRA* and CΔ*cpxRA* strains with or without 0.3 M K^+^ or 0.3 M Na^+^ were monitored by measurement of spotting on TSA plates **(A)**, OD600 **(B)**, and viable cell counts **(C)**. ^**^p < 0.01. ^***^p < 0.001.

In the published article, there was an error in the legend for **Figure 2** as published. The concentration of potassium ions was reported incorrectly. The corrected legend appears below.

Figure 2 CpxAR impacts cell division under high-K^+^ stress. **(A)** Observation of bacterial length in the WT, Δ*cpxRA*, and CΔ*cpxRA* strains grown with or without 0.3 M K^+^ by SEM. **(B)** Bacterial length measured for the WT, Δ*cpxRA* and CΔ*cpxRA* strains grown with or without 0.3 M K^+^. The number of bacteria measured is shown in brackets. ^***^p < 0.001.

In the published article, there was an error. The concentration of KCl added to the TSB was reported incorrectly.

A correction has been made to **Materials and methods**, *SEM*. This sentence previously stated:

“For scanning electron microscopy (SEM), wild-type S4074 and its mutant derivatives were harvested after they were cultured in TSB medium supplemented with 10% FBS and 10% NAD with or without the supplementation of 0.3 mM KCl. Bacterial cells were fixed with 2.5% glutaraldehyde and deposited onto copper grids (200 mesh; Zhongjingkeyi, China). The copper grids were air-dried, mounted on the sample stub and coated with gold. Subsequently, strains were observed by the SEM (VEGA3; TESCAN, Czech) and the bacterial length was measured by Image J (NIH, United States).”

The corrected sentence appears below:

“For scanning electron microscopy (SEM), wild-type S4074 and its mutant derivatives were harvested after they were cultured in TSB medium supplemented with 10% FBS and 10% NAD with or without the supplementation of 0.3 M KCl. Bacterial cells were fixed with 2.5% glutaraldehyde and deposited onto copper grids (200 mesh; Zhongjingkeyi, China). The copper grids were air-dried, mounted on the sample stub and coated with gold. Subsequently, strains were observed by the SEM (VEGA3; TESCAN, Czech) and the bacterial length was measured by Image J (NIH, United States).”

In the published article, there was an error. The concentration of KCl added to the TSB was reported incorrectly.

A correction has been made to **Materials and methods**, *Construction of promoter-gfp repoter strains and promoter analyses*. This sentence previously stated:

“A *ftsE*-*gfp* fusion containing the *ftsE* promoter region and *gfp* gene, was cloned into the Xho I and Not I sites on the pJFF224-XN plasmid. Then, the *ftsE*-*gfp* reporter plasmid was electroporated into the wild-type S4074 and the Δ*cpxAR* mutant strain. *A. pleuropneumoniae* strains harboring a *ftsE*-*gfp* reporter plasmid were grown to an OD600 of 0.6 in TSB medium with or without the supplementation of 0.3 mM KCl. The cells were harvested and resuspended in 1 mL of 10 mM PBS. Luminescence was measured in the Spectramax iD3 microplate reader with excitation at 485 nm and emission at 535 nm.”

The corrected sentence appears below:

“A *ftsE*-*gfp* fusion containing the *ftsE* promoter region and *gfp* gene, was cloned into the Xho I and Not I sites on the pJFF224-XN plasmid. Then, the *ftsE*-*gfp* reporter plasmid was electroporated into the wild-type S4074 and the Δ*cpxAR* mutant strain. *A. pleuropneumoniae* strains harboring a *ftsE*-*gfp* reporter plasmid were grown to an OD600 of 0.6 in TSB medium with or without the supplementation of 0.3 M KCl. The cells were harvested and resuspended in 1 mL of 10 mM PBS. Luminescence was measured in the Spectramax iD3 microplate reader with excitation at 485 nm and emission at 535 nm.”

In the published article, there was an error. The concentrations of KCl and NaCl were reported incorrectly.

A correction has been made to **Results**, *Deletion of the cpxRA genes decreases the growth of Actinobacillus pleuropneumoniae under high-K*^+^ *stress*. This sentence previously stated:

“To explore the role of CpxAR in bacterial adaptation to osmotic stress in *A. pleuropneumoniae*, we tested the growth traits of the wild-type S4074 and its *cpxRA* mutant strain grown in solid or liquid medium with or without the supplementation of 0.3 mM KCl or 0.3 mM NaCl. When the cells were grown in solid medium, the growth of the Δ*cpxRA* strain was markedly reduced with the supplementation of 0.3 mM KCl, but similar to that of the WT strain with the supplementation of 0.3 mM NaCl or normal medium (Figure 1A). Optical density and colony forming units showed that the growth rate of the Δ*cpxRA* strain was significantly lower than that of the WT strain when grown with the supplementation of 0.3 mM KCl (Figures 1B, C). These findings suggested that the growth defect of the mutant strain Δ*cpxRA* was significantly increased compared with that of WT and CΔ*cpxRA* strains under high-K^+^ stress. These results suggested that CpxAR contributes to *A. pleuropneumoniae* survival under high potassium stress.”

The corrected sentence appears below:

“To explore the role of CpxAR in bacterial adaptation to osmotic stress in *A. pleuropneumoniae*, we tested the growth traits of the wild-type S4074 and its *cpxRA* mutant strain grown in solid or liquid medium with or without the supplementation of 0.3 M KCl or 0.3 M NaCl. When the cells were grown in solid medium, the growth of the Δ*cpxRA* strain was markedly reduced with the supplementation of 0.3 M KCl, but similar to that of the WT strain with the supplementation of 0.3 M NaCl or normal medium (Figure 1A). Optical density and colony forming units showed that the growth rate of the Δ*cpxRA* strain was significantly lower than that of the WT strain when grown with the supplementation of 0.3 M KCl (Figures 1B, C). These findings suggested that the growth defect of the mutant strain Δ*cpxRA* was significantly increased compared with that of WT and CΔ*cpxRA* strains under high-K^+^ stress. These results suggested that CpxAR contributes to *A. pleuropneumoniae* survival under high potassium stress.”

In the published article, there was an error. The concentration of KCl was reported incorrectly.

A correction has been made to **Results**, *Deletion of cpxRA affects the cell division of Actinobacillus pleuropneumoniae under high-K*^+^ *stress*. This sentence previously stated:

“To further investigate how the inactivation of *cpxRA* genes affected the growth of *A. pleuropneumoniae* under high-K^+^ stress, we used scanning electron microscope (SEM) to observe the bacterial morphology of the WT, Δ*cpxRA* and CΔ*cpxRA* strains when they were grown with or without the supplementation of 0.3 mM KCl. When grown with the supplementation of 0.3 mM KCl, the cell length of Δ*cpxRA* strain showed a 2-fold increase on average compared to the wild-type and CΔ*cpxRA* strains (Figures 2A, B). In addition, we found that the cell length of Δ*cpxRA* strain was also longer than that of the wild-type and CΔ*cpxRA* strains without K^+^ stress, but the difference was much smaller than that under K^+^ stress (Figures 2A, B). Together, these findings suggested that CpxAR regulates cell division to help *A. pleuropneumoniae* cope with potassium stress.”

The corrected sentence appears below:

“To further investigate how the inactivation of *cpxRA* genes affected the growth of *A. pleuropneumoniae* under high-K^+^ stress, we used scanning electron microscope (SEM) to observe the bacterial morphology of the WT, Δ*cpxRA* and CΔ*cpxRA* strains when they were grown with or without the supplementation of 0.3 M KCl. When grown with the supplementation of 0.3 M KCl, the cell length of Δ*cpxRA* strain showed a two-fold increase on average compared to the wild-type and CΔ*cpxRA* strains (Figures 2A, B). In addition, we found that the cell length of Δ*cpxRA* strain was also longer than that of the wild-type and CΔ*cpxRA* strains without K^+^ stress, but the difference was much smaller than that under K^+^ stress (Figures 2A, B). Together, these findings suggested that CpxAR regulates cell division to help *A. pleuropneumoniae* cope with potassium stress.”

In the published article, there was an error. The concentration of KCl was reported incorrectly.

A correction has been made to **Results**, *CpxAR regulates the expression of cell division genes ftsE and ftsX under high-K*^+^ *stress*, Paragraph 1. This sentence previously stated:

“To gain insight into the mechanism of CpxAR affecting the cell division of *A. pleuropneumoniae*, we compared the transcript levels of cell division genes in the WT and Δ*cpxRA* strains by qRT-PCR. As shown in Figure 3A, the relative transcript levels of *ftsE* and *ftsX* were significantly downregulated in the Δ*cpxRA* strain with or without the supplementation of 0.3 mM KCl, but *ftsY* was not (Figure 3A). However, there were no significant changes in the cell division genes *ftsL, ftsI, ftsW, ftsQ, ftsZ, ftsB, ftsA, zipA, ftsH*, and *ftsK* in the Δ*cpxRA* strain compared with the WT strain (Supplementary Figure S1). These results suggested that CpxAR regulates the expression of the cell division genes *ftsE* and *ftsX* in *A. pleuropneumoniae*.”

The corrected sentence appears below:

“To gain insight into the mechanism of CpxAR affecting the cell division of *A. pleuropneumoniae*, we compared the transcript levels of cell division genes in the WT and Δ*cpxRA* strains by qRT-PCR. As shown in Figure 3A, the relative transcript levels of *ftsE* and *ftsX* were significantly downregulated in the Δ*cpxRA* strain with or without the supplementation of 0.3 M KCl, but *ftsY* was not (Figure 3A). However, there were no significant changes in the cell division genes *ftsL, ftsI, ftsW, ftsQ, ftsZ, ftsB, ftsA, zipA, ftsH*, and *ftsK* in the Δ*cpxRA* strain compared with the WT strain (Supplementary Figure S1). These results suggested that CpxAR regulates the expression of the cell division genes *ftsE* and *ftsX* in *A. pleuropneumoniae*.”

The original article has been updated.

