# Supplementary figures and images for: The two-component system CpxAR is required for the high potassium stress survival of Actinobacillus pleuropneumoniae
Source: Front Microbiol. 2023 Sep 26;14:1259935. doi: 10.3389/fmicb.2023.1259935 (PMC10562621; doi:10.3389/fmicb.2023.1259935)

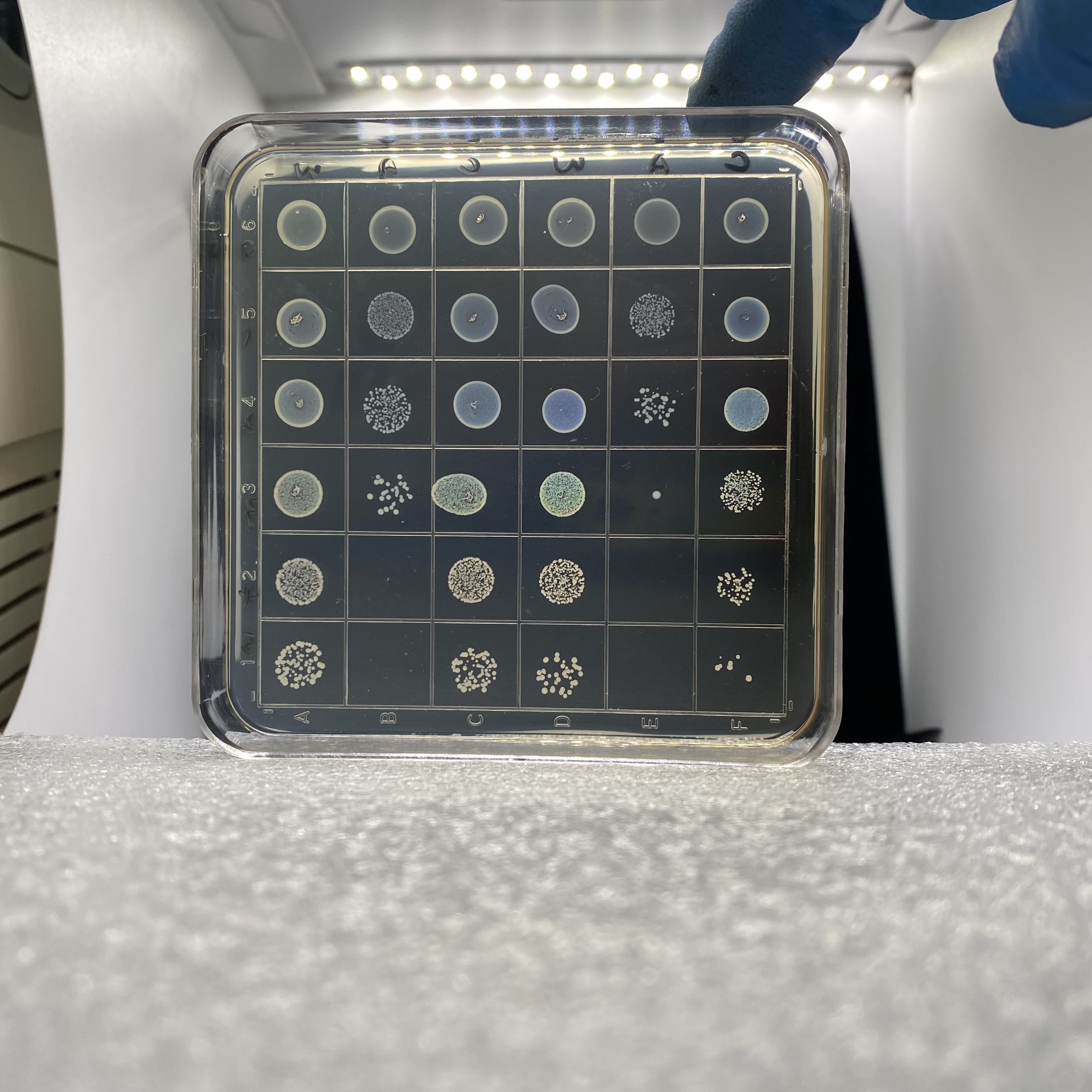

Supplement: Supplementary file 1 [file Data_Sheet_1.ZIP › original data/original data/figure 1/figure 1-A/0.3 M KCl.jpg]

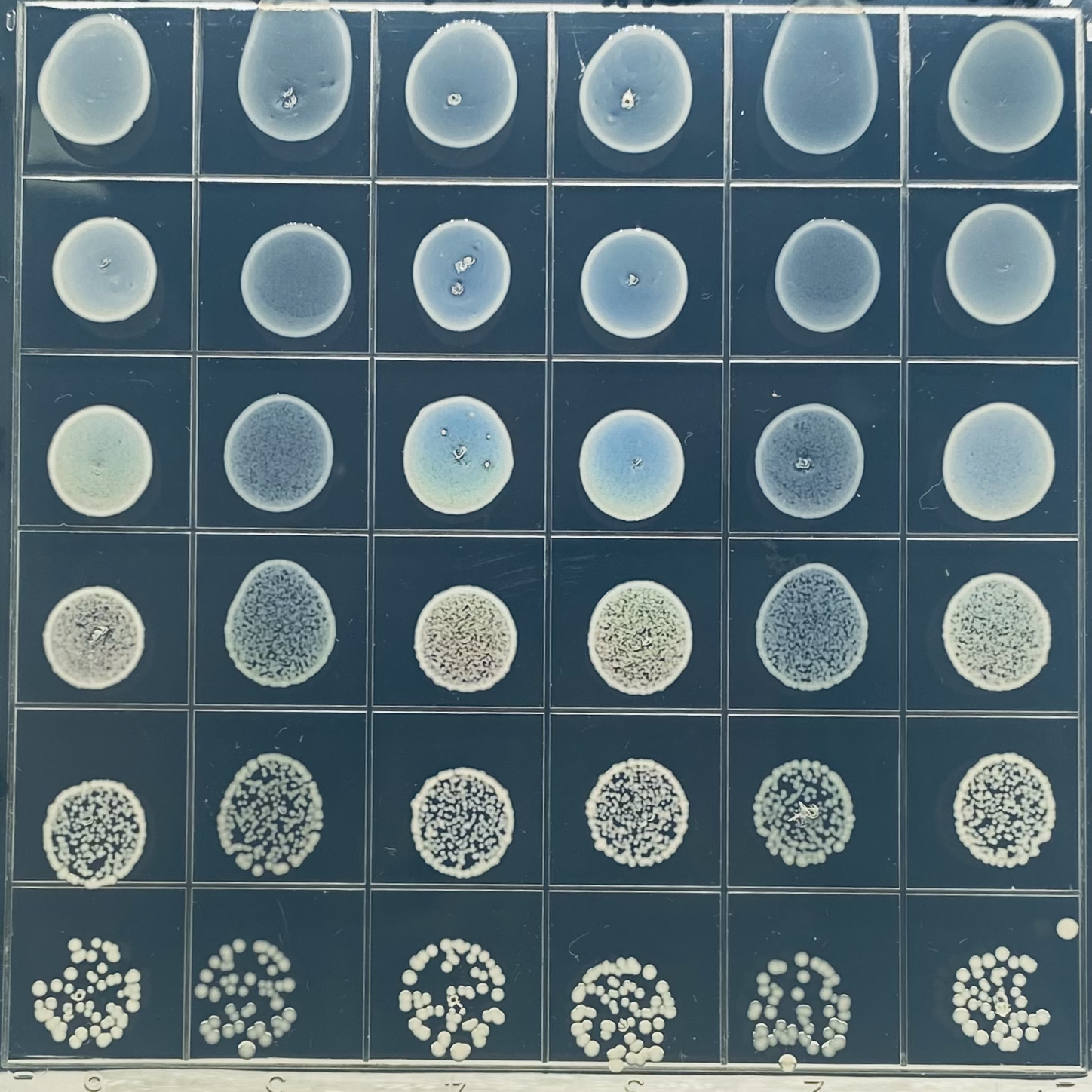

Supplement: Supplementary file 1 [file Data_Sheet_1.ZIP › original data/original data/figure 1/figure 1-A/0.3M NaCL.jpg]

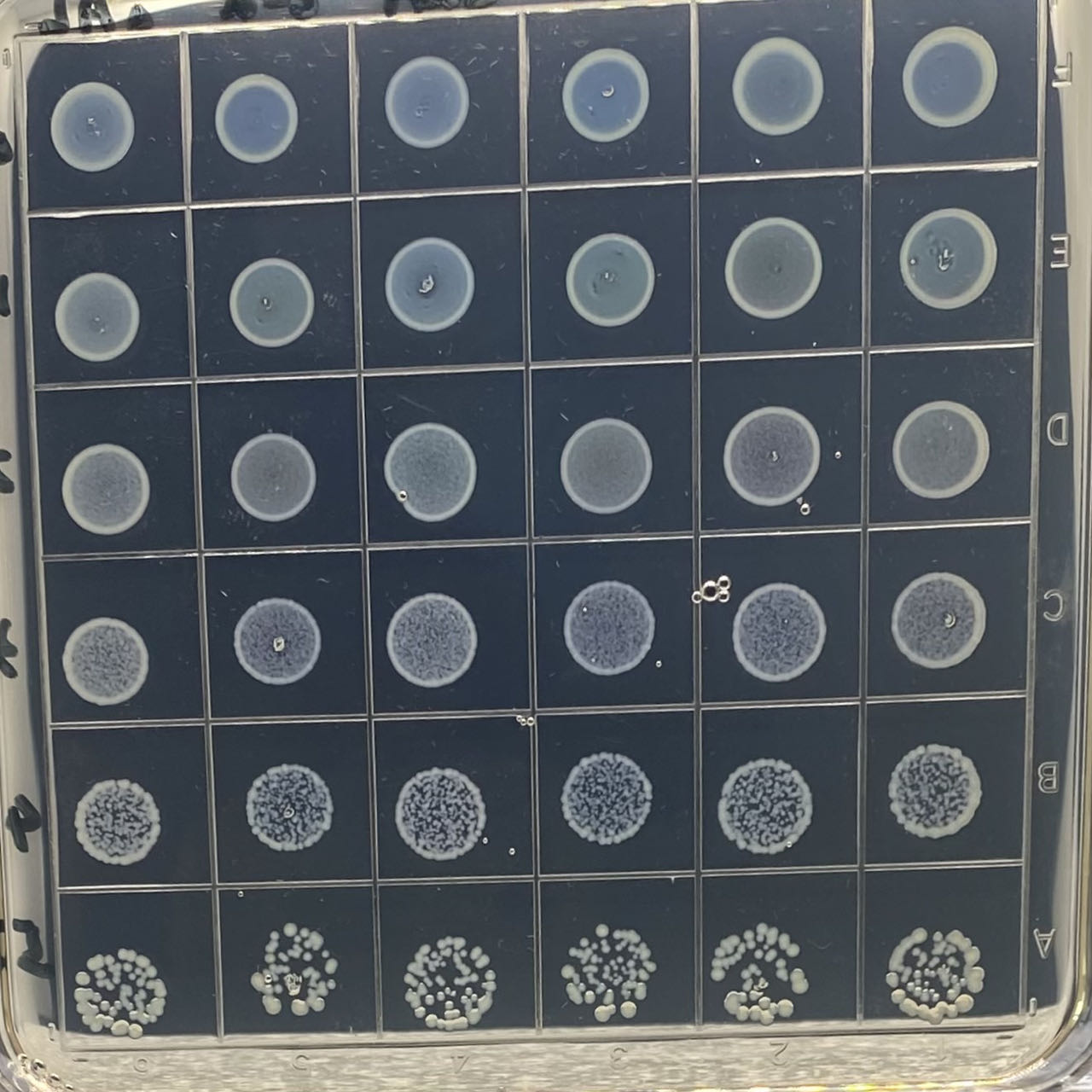

Supplement: Supplementary file 1 [file Data_Sheet_1.ZIP › original data/original data/figure 1/figure 1-A/control.jpg]

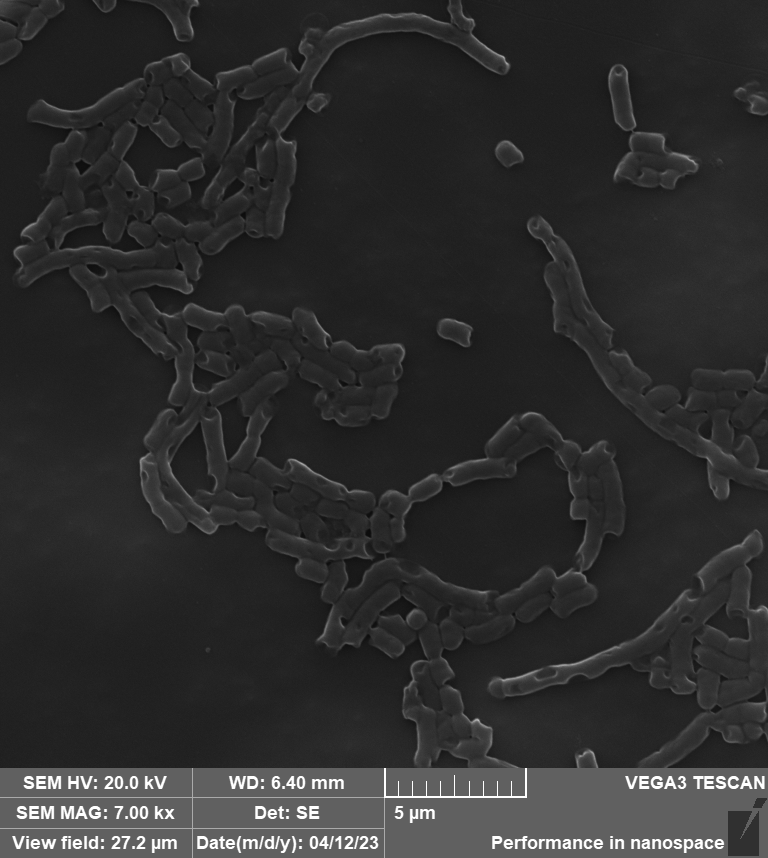

Supplement: Supplementary file 1 [file Data_Sheet_1.ZIP › original data/original data/figure 2/figure 2-A/0.3M K+/C-cpxRA-K+.tif]

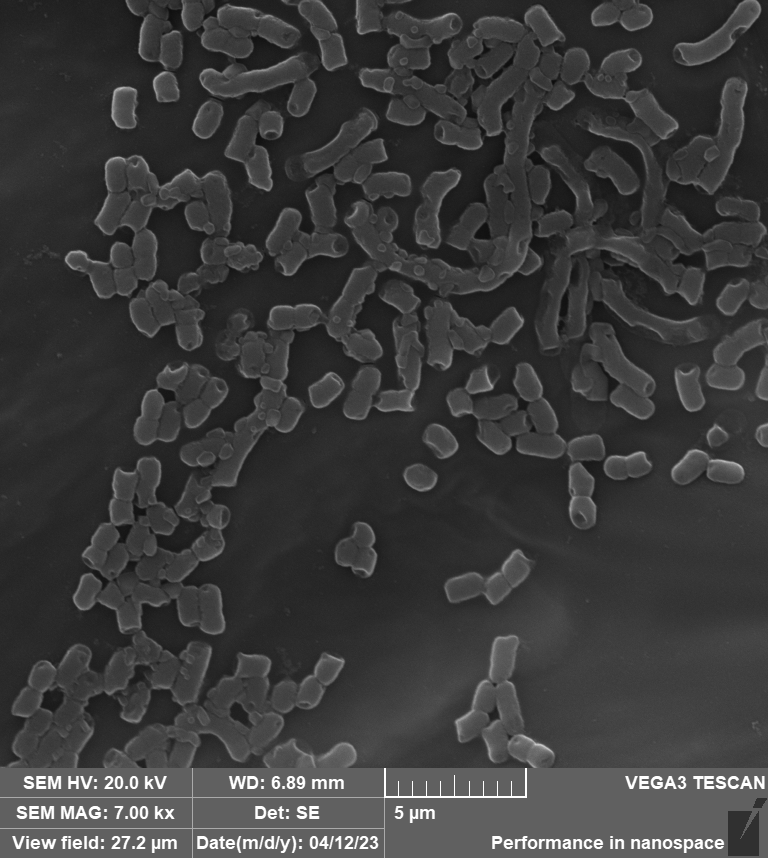

Supplement: Supplementary file 1 [file Data_Sheet_1.ZIP › original data/original data/figure 2/figure 2-A/0.3M K+/WT-K+.tif]

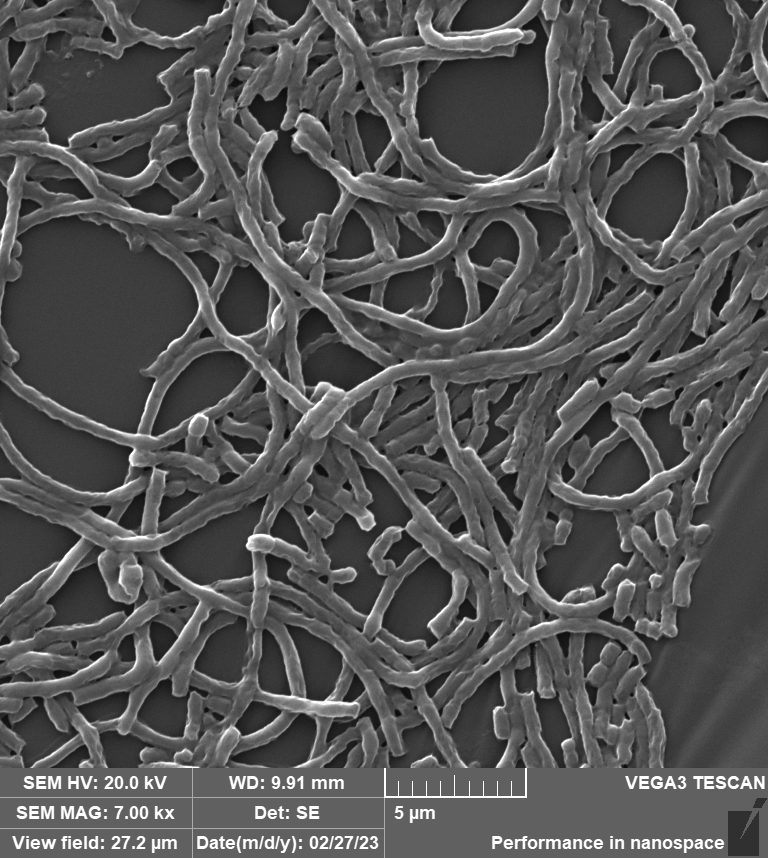

Supplement: Supplementary file 1 [file Data_Sheet_1.ZIP › original data/original data/figure 2/figure 2-A/0.3M K+/cpxRA-K+.tif]

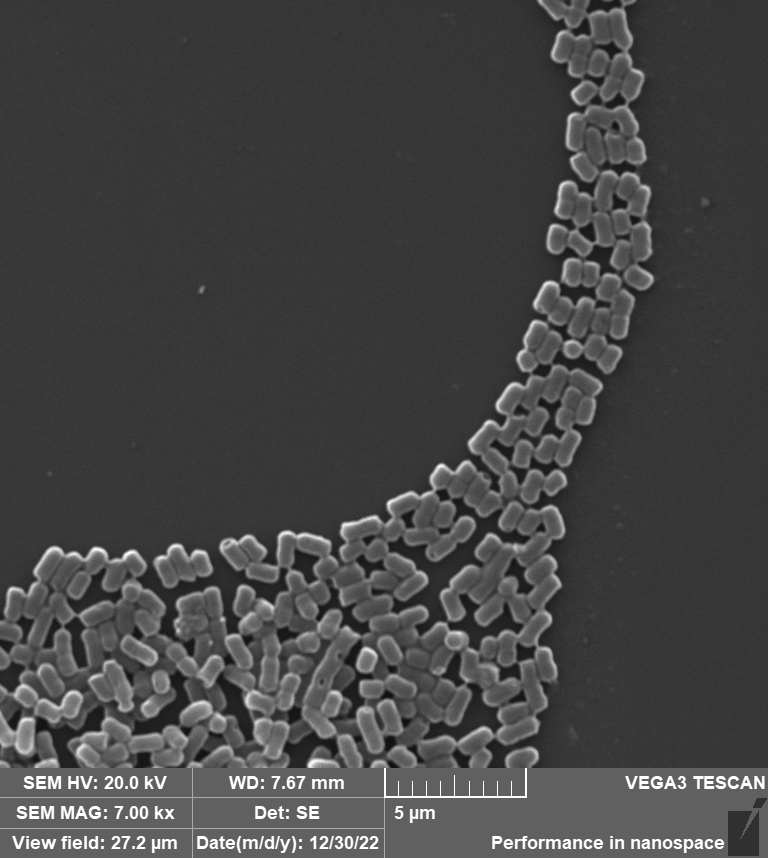

Supplement: Supplementary file 1 [file Data_Sheet_1.ZIP › original data/original data/figure 2/figure 2-A/control/C-cpxRA.tif]

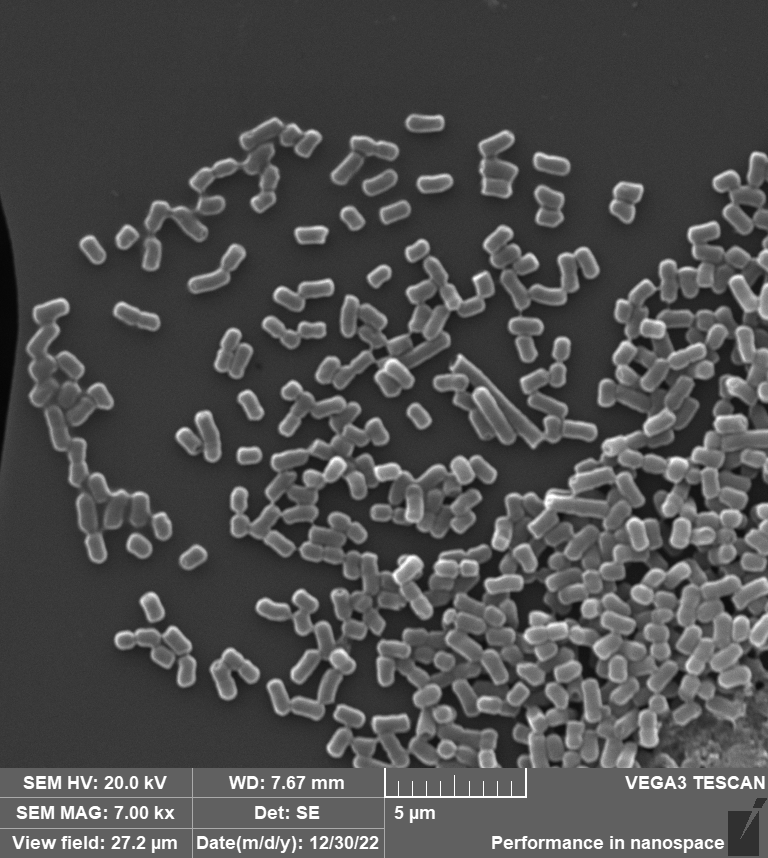

Supplement: Supplementary file 1 [file Data_Sheet_1.ZIP › original data/original data/figure 2/figure 2-A/control/WT.tif]

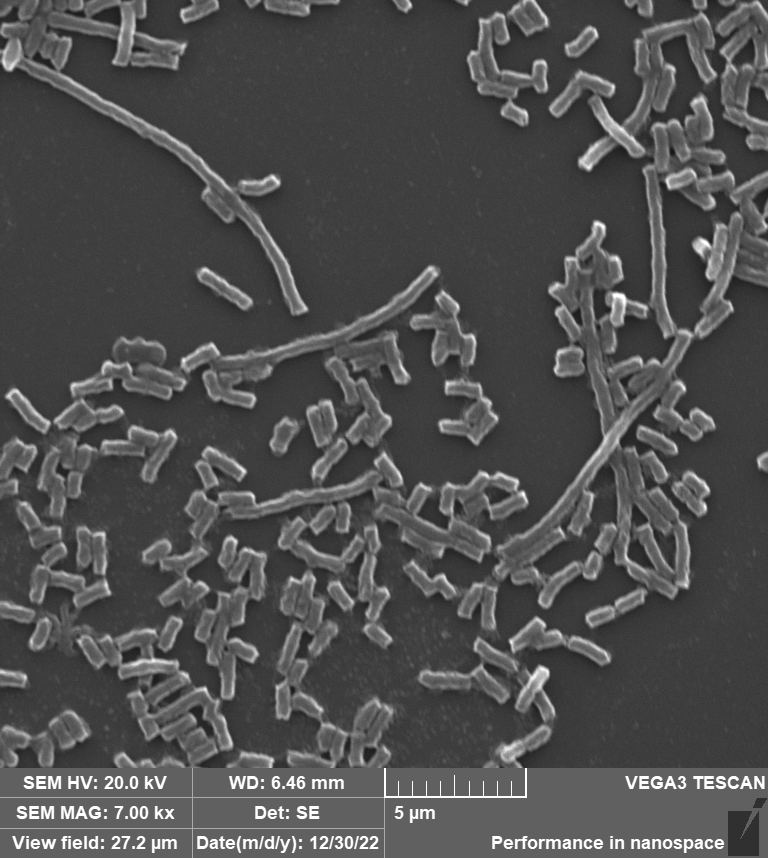

Supplement: Supplementary file 1 [file Data_Sheet_1.ZIP › original data/original data/figure 2/figure 2-A/control/cpxRA.tif]

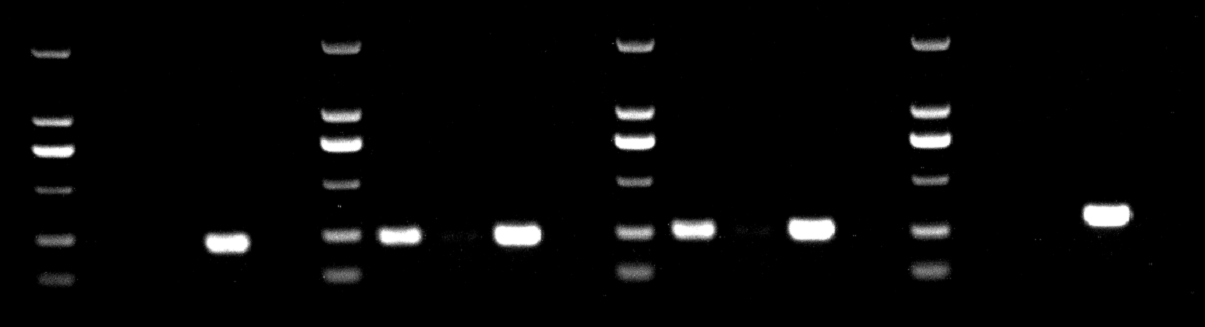

Supplement: Supplementary file 1 [file Data_Sheet_1.ZIP › original data/original data/figure 3/figure 3-B/RT-PCR-1.tif]

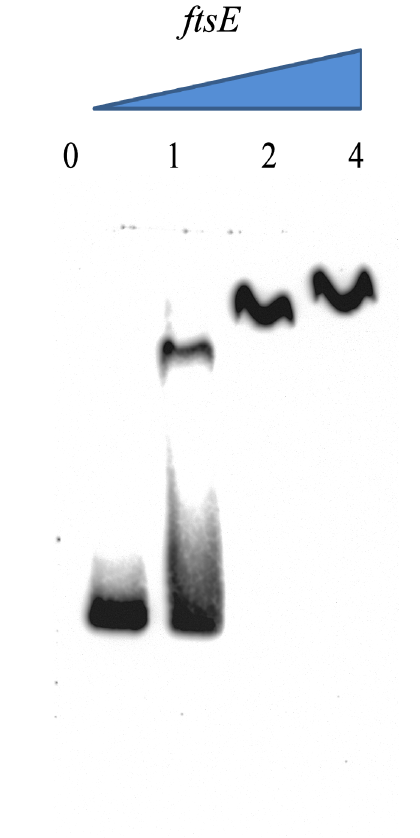

Supplement: Supplementary file 1 [file Data_Sheet_1.ZIP › original data/original data/figure 3/figure 3-D/ftsE.tif]

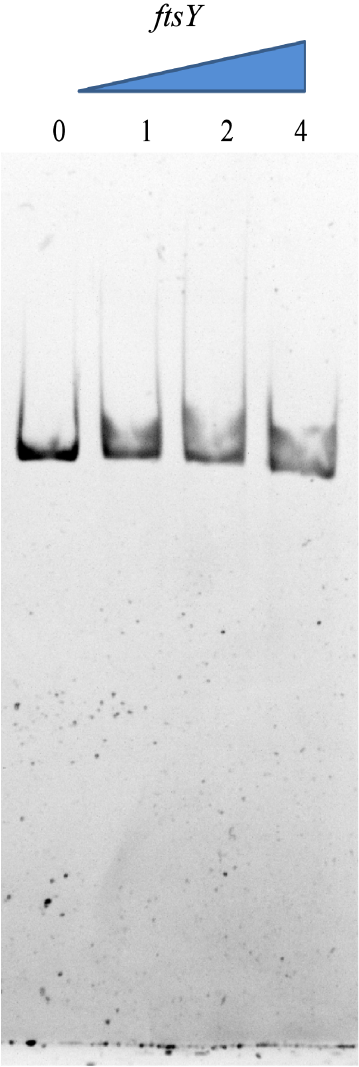

Supplement: Supplementary file 1 [file Data_Sheet_1.ZIP › original data/original data/figure 3/figure 3-D/ftsY.tif]

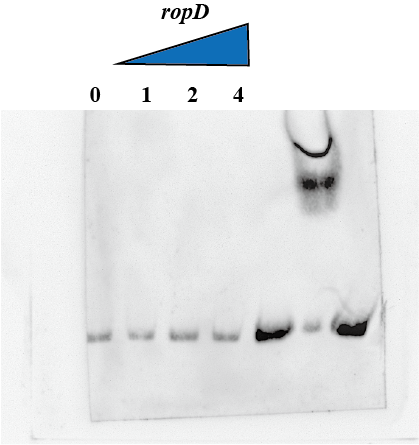

Supplement: Supplementary file 1 [file Data_Sheet_1.ZIP › original data/original data/figure 3/figure 3-D/ropD.tif]

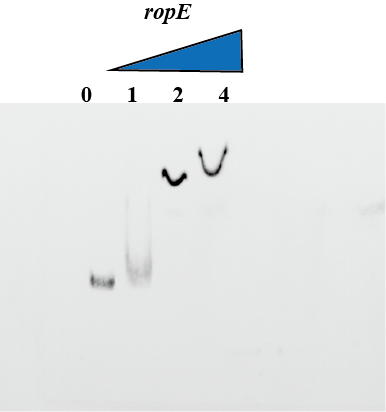

Supplement: Supplementary file 1 [file Data_Sheet_1.ZIP › original data/original data/figure 3/figure 3-D/ropE.tif]

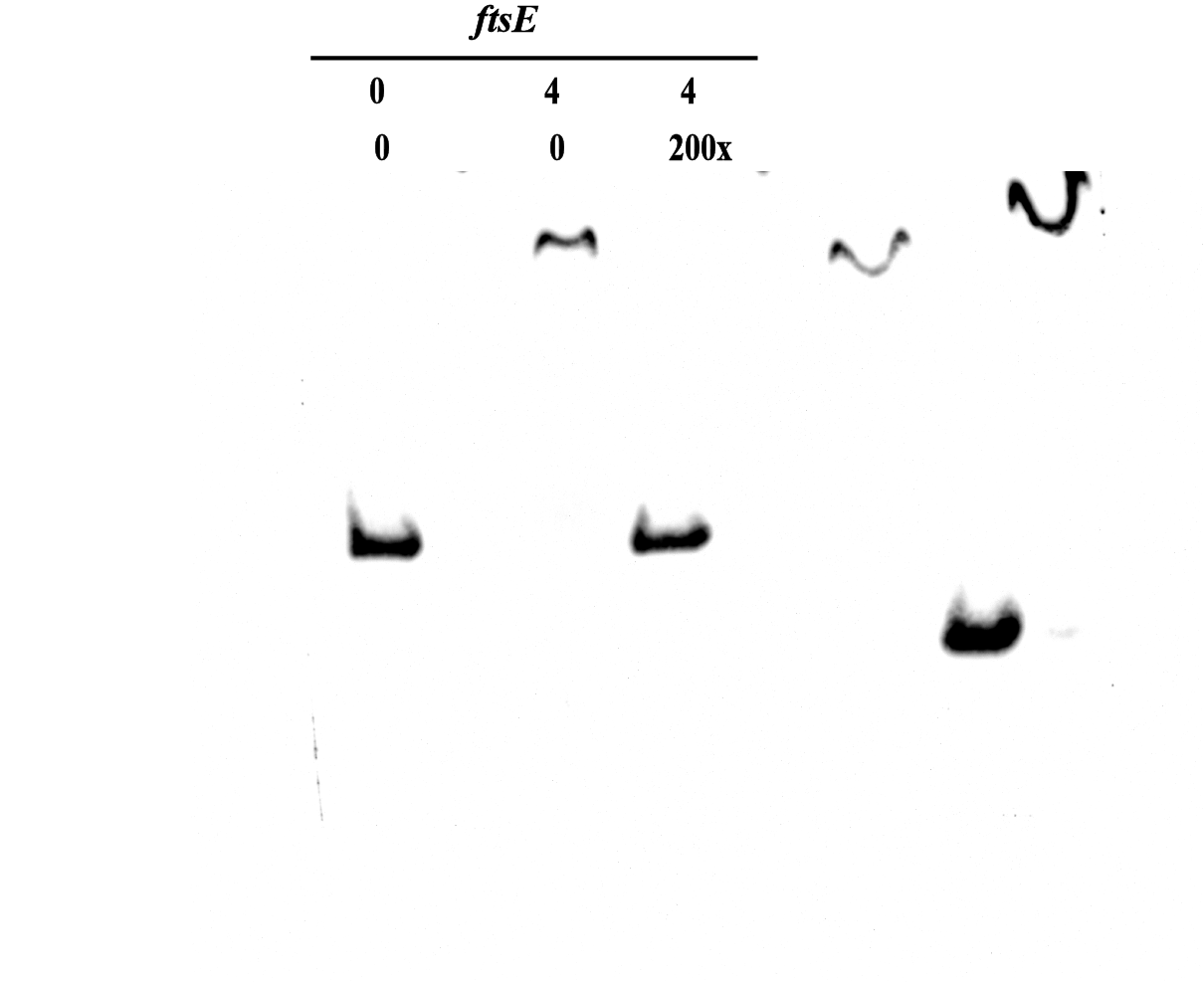

Supplement: Supplementary file 1 [file Data_Sheet_1.ZIP › original data/original data/figure 3/figure 3-E/ftsE竞争.tif]

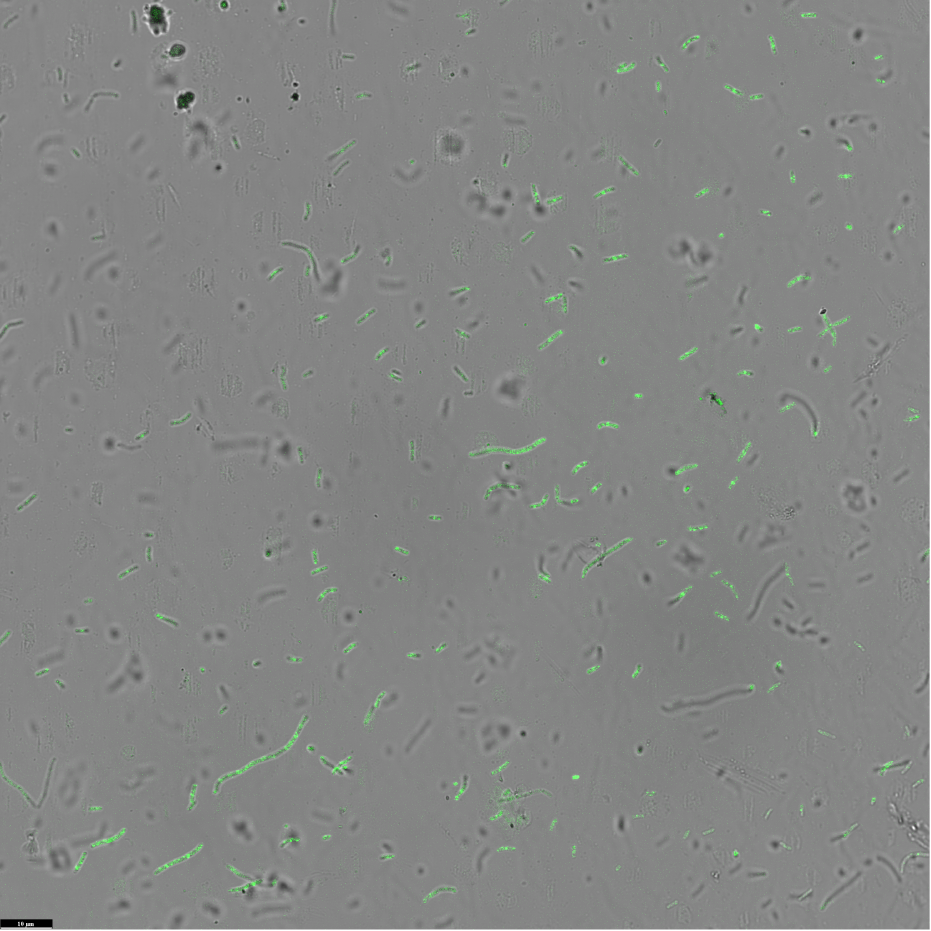

Supplement: Supplementary file 1 [file Data_Sheet_1.ZIP › original data/original data/figure 3/figure 3-G/0.3M K+/C-cpxAR/C-cpxAR-1.tif]

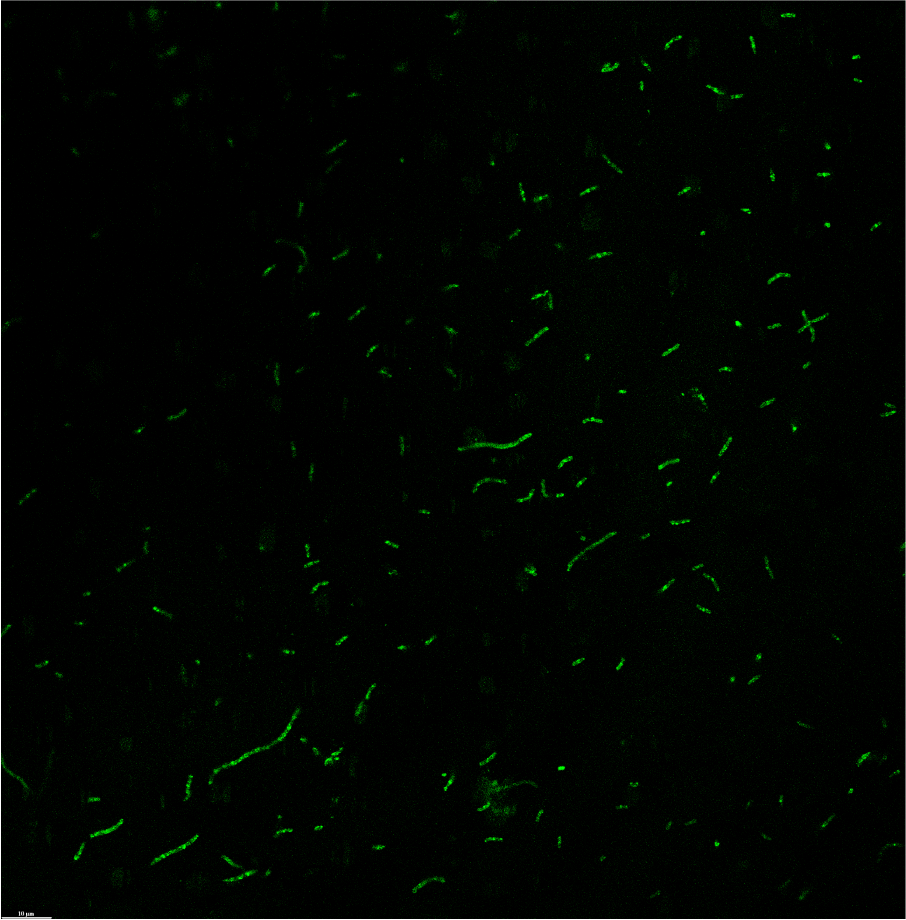

Supplement: Supplementary file 1 [file Data_Sheet_1.ZIP › original data/original data/figure 3/figure 3-G/0.3M K+/C-cpxAR/C-cpxAR-2.tif]

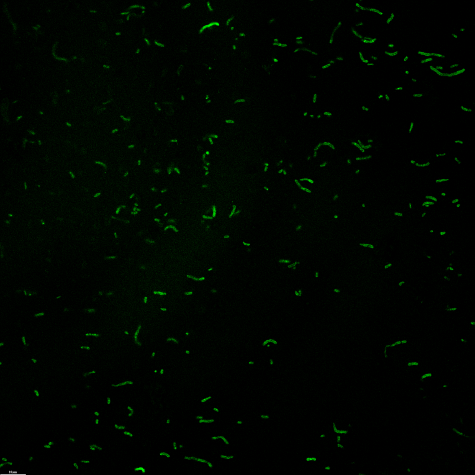

Supplement: Supplementary file 1 [file Data_Sheet_1.ZIP › original data/original data/figure 3/figure 3-G/0.3M K+/WT/WT -1.tif]

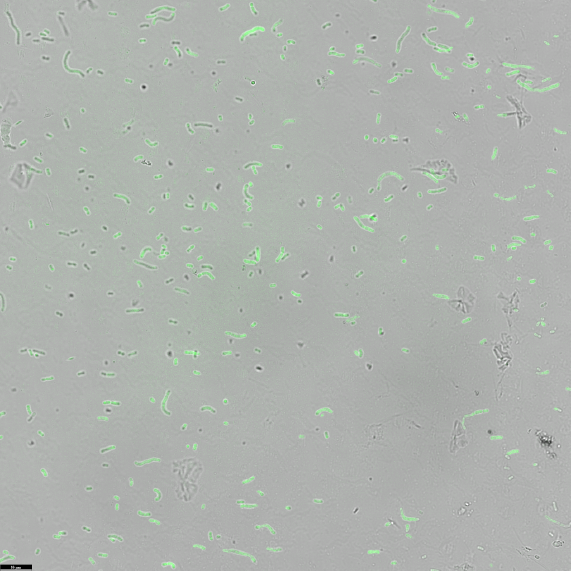

Supplement: Supplementary file 1 [file Data_Sheet_1.ZIP › original data/original data/figure 3/figure 3-G/0.3M K+/WT/WT-2.tif]

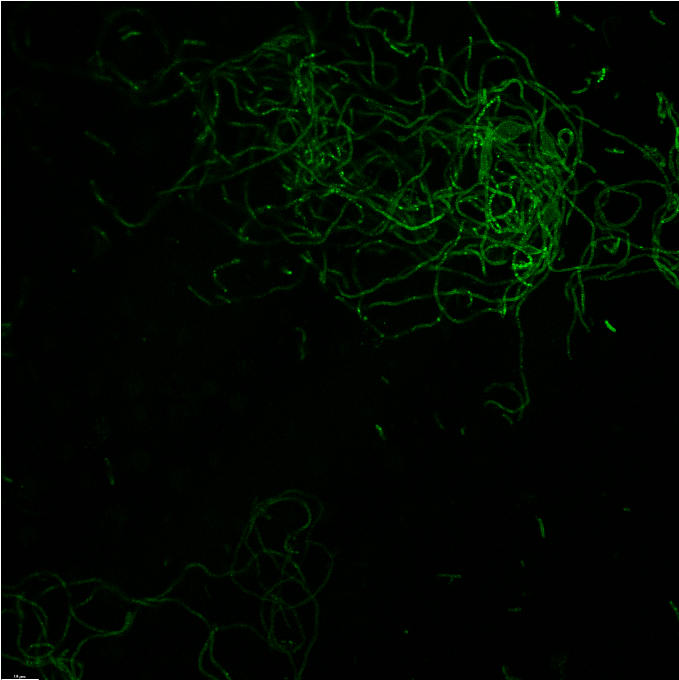

Supplement: Supplementary file 1 [file Data_Sheet_1.ZIP › original data/original data/figure 3/figure 3-G/0.3M K+/cpxAR/cpxAR-1.tif]

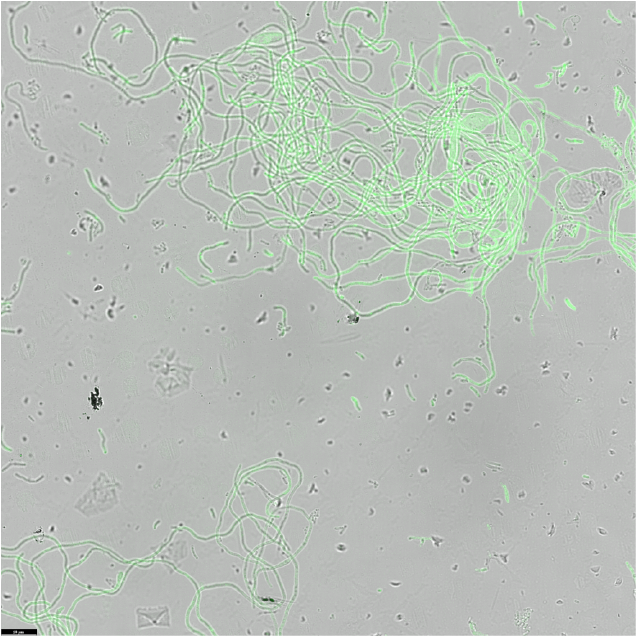

Supplement: Supplementary file 1 [file Data_Sheet_1.ZIP › original data/original data/figure 3/figure 3-G/0.3M K+/cpxAR/cpxAR-2.tif]

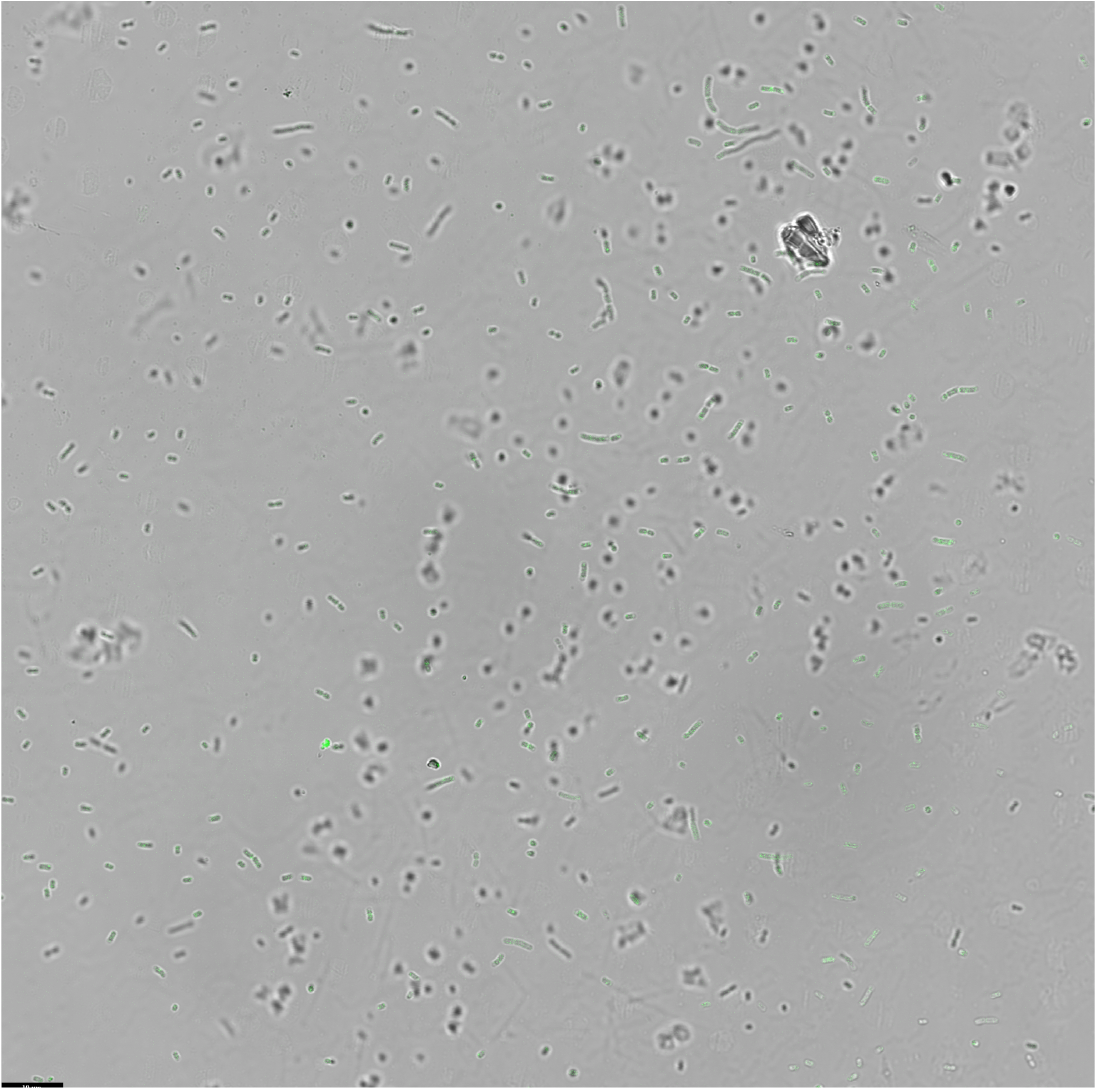

Supplement: Supplementary file 1 [file Data_Sheet_1.ZIP › original data/original data/figure 3/figure 3-G/control/C-cpxRA/C-cpxAR-1.tif]

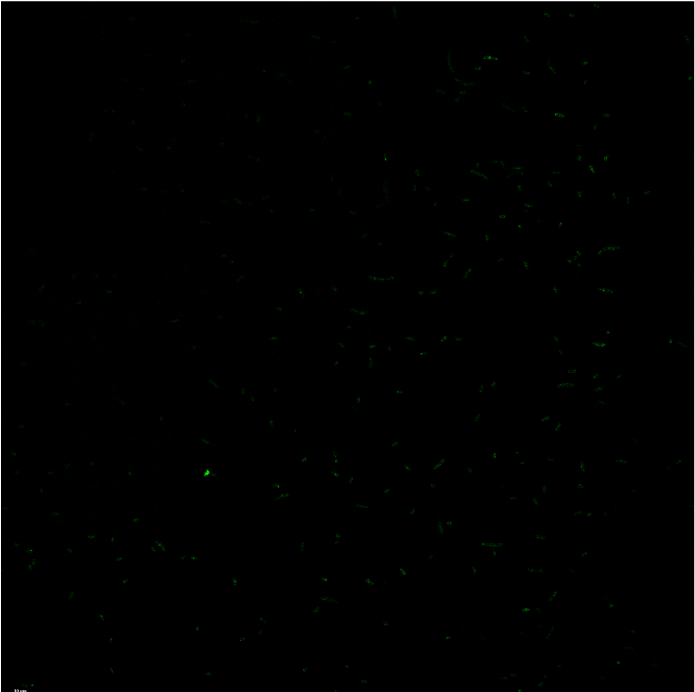

Supplement: Supplementary file 1 [file Data_Sheet_1.ZIP › original data/original data/figure 3/figure 3-G/control/C-cpxRA/C-cpxRA-2.tif]

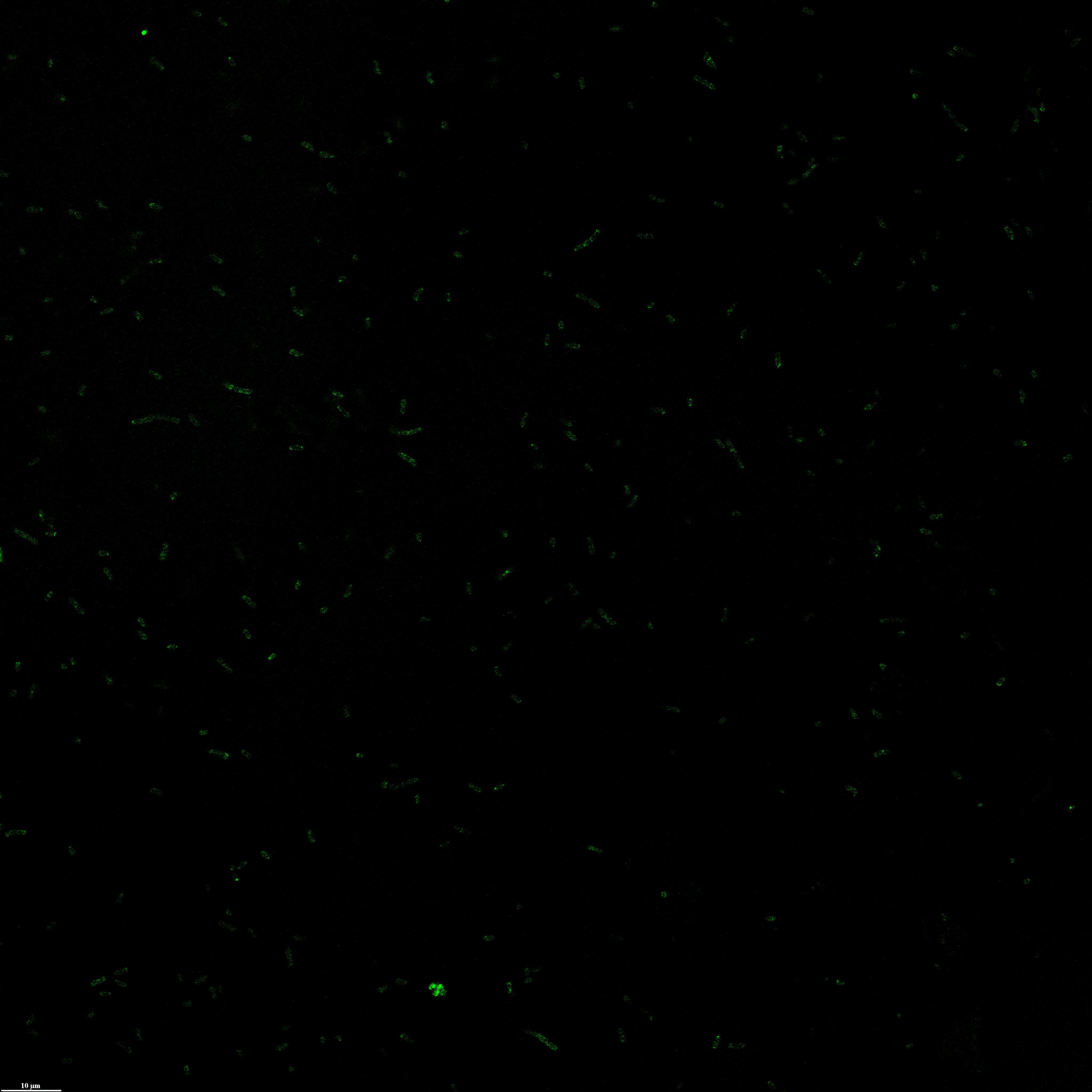

Supplement: Supplementary file 1 [file Data_Sheet_1.ZIP › original data/original data/figure 3/figure 3-G/control/WT/WT-1.tif]

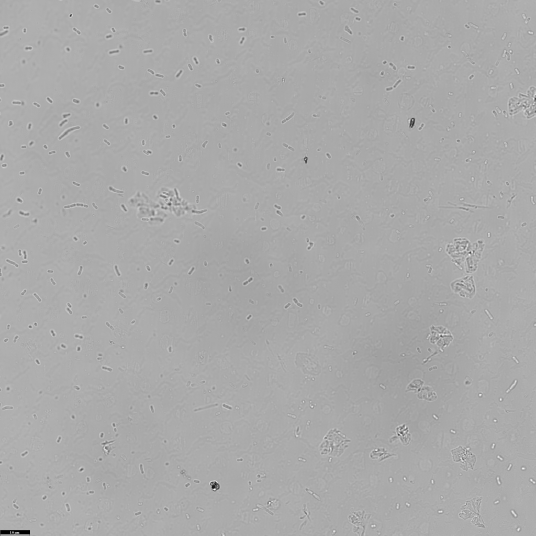

Supplement: Supplementary file 1 [file Data_Sheet_1.ZIP › original data/original data/figure 3/figure 3-G/control/WT/WT-2.tif]

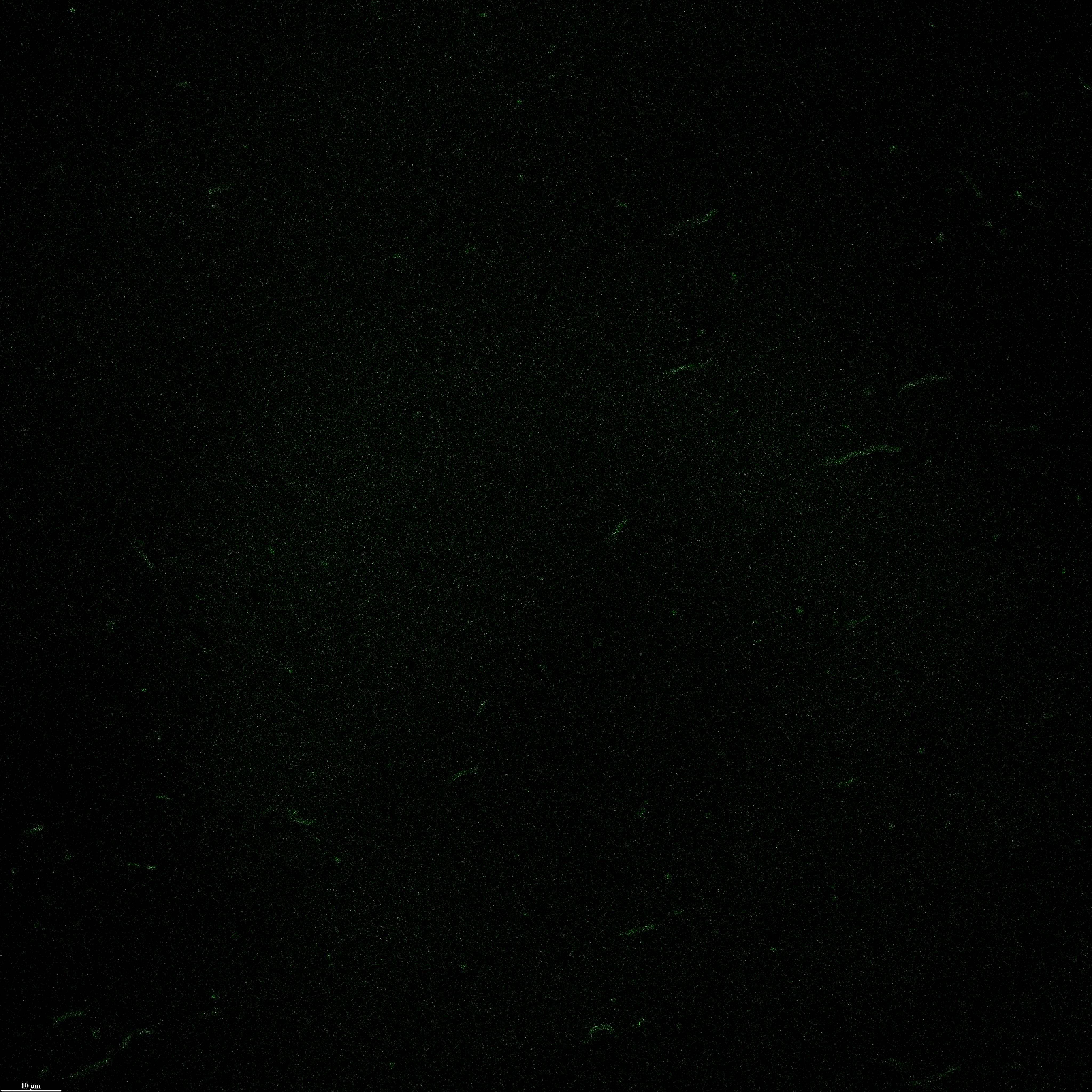

Supplement: Supplementary file 1 [file Data_Sheet_1.ZIP › original data/original data/figure 3/figure 3-G/control/cpxRA/cpxRA-1.tif]

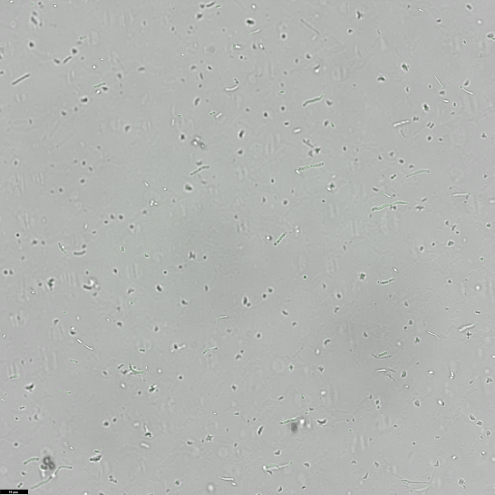

Supplement: Supplementary file 1 [file Data_Sheet_1.ZIP › original data/original data/figure 3/figure 3-G/control/cpxRA/cpxRA-2.tif]

## Slide 1
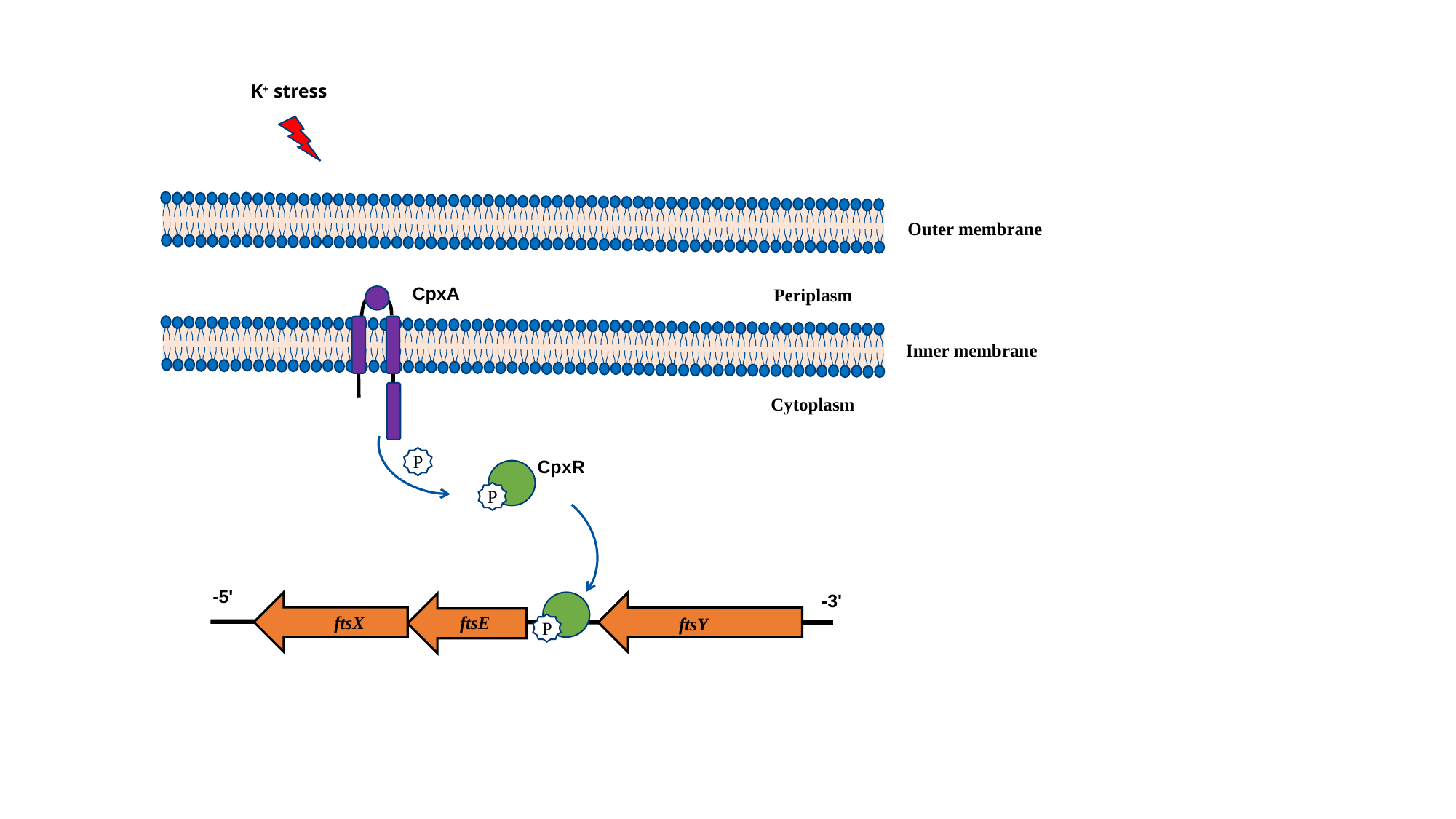

K+ stress
Outer membrane
CpxA
Periplasm
Inner membrane
Cytoplasm
P
CpxR
P
-5'
-3'
ftsX
ftsE
ftsY
P

Supplement: Supplementary file 1 [file Data_Sheet_1.ZIP › original data/original data/figure-4/figure-5.pptx]

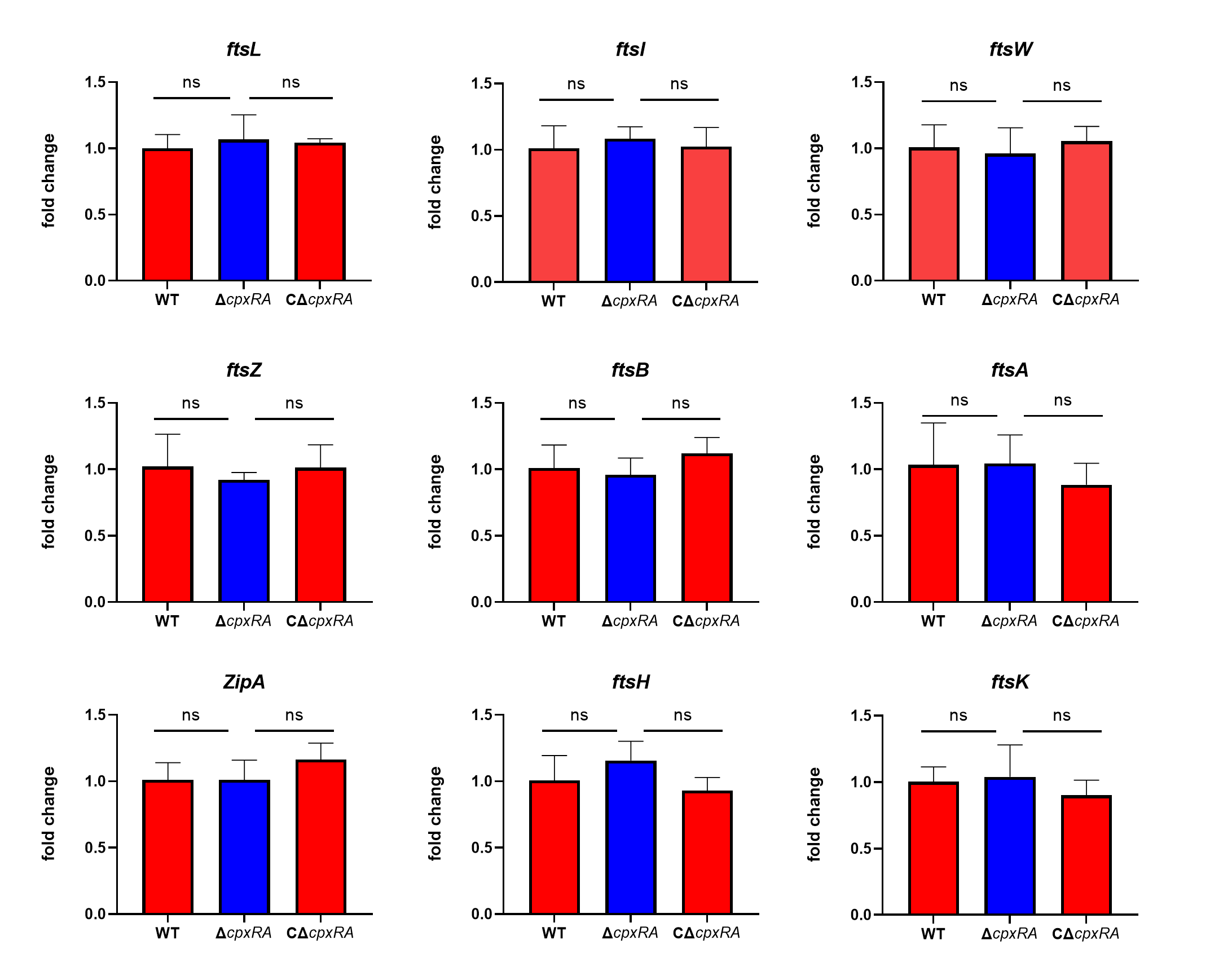

Supplement: Supplementary file 2 [file Image_1.tif]
